# The Cortico-Limbo-Thalamo-Cortical Circuits: An Update to the Original Papez Circuit of the Human Limbic System

**DOI:** 10.1007/s10548-023-00955-y

**Published:** 2023-04-26

**Authors:** Arash Kamali, Sofia Milosavljevic, Anusha Gandhi, Kinsey R. Lano, Parnian Shobeiri, Farzaneh Ghazi Sherbaf, Haris I. Sair, Roy F. Riascos, Khader M. Hasan

**Affiliations:** 1 Department of Diagnostic and Interventional Radiology, Neuroradiology Section, University of Texas at Houston, 6431 Fannin St, Houston, TX 77030 USA; 2 Harvard Medical School, Boston, MA USA; 3 Baylor College of Medicine Medical School, Houston, TX USA; 4 McGovern Medical School, University of Texas Health Science Center at Houston, Houston, TX USA; 5 Faculty of Medicine, Tehran University Medical School, Tehran, Iran; 6 Department of Radiology and Radiological Science, Division of Neuroradiology, The Russell H. Morgan, Johns Hopkins University, Baltimore, MD USA

**Keywords:** Limbic, Tractography, White matter connections, Brain networks, Diffusion-weighted imaging, Papez circuit

## Abstract

The Papez circuit, first proposed by James Papez in 1937, is a circuit believed to control memory and emotions, composed of the cingulate cortex, entorhinal cortex, parahippocampal gyrus, hippocampus, hypothalamus, and thalamus. Pursuant to James Papez, Paul Yakovlev and Paul MacLean incorporated the prefrontal/orbitofrontal cortex, septum, amygdalae, and anterior temporal lobes into the limbic system. Over the past few years, diffusion-weighted tractography techniques revealed additional limbic fiber connectivity, which incorporates multiple circuits to the already known complex limbic network. In the current review, we aimed to comprehensively summarize the anatomy of the limbic system and elaborate on the anatomical connectivity of the limbic circuits based on the published literature as an update to the original Papez circuit.

**Supplementary Information:**

The online version contains supplementary material available at 10.1007/s10548-023-00955-y.

## Introduction

The word limbic (Latin word for border) first introduced by Thomas Willis designating a cortical border structure encircling the brainstem (Thomas Willis 1664). Broca proposed olfactory structures to be the main components of the limbic system in mammalian brains (Broca 1878). Later, Christopher Jakob (Christopher Jakob 1906) and James Papez (James Papez 1937) introduced the limbic system as an integrated system of cortical and subcortical structures involved in linking actions and perceptions to emotions. The Papez circuit, first proposed by James Papez in 1937, is a major pathway of the limbic system and is believed to be involved in controlling both memory and emotion (Papez 1937; Roxo et al. 2011). Papez first proposed the idea of a link between the hippocampus, hypothalamus (mammillary bodies), thalamus, cingulate gyrus, and parahippocampal gyrus (Papez 1937). Papez further integrated the anatomical definition of limbic models into a functional model. According to Papez, two circuits are involved in the emotional process: one the medial circuit via the hippocampus and cingulate cortex, which are directly involved with the hypothalamus, the other via the lateral cortex, which is involved in sensory activities mediated by the dorsal thalamus (Papez 1937). Paul Yakovlev introduced the basolateral circuit, including the thalamo-amygdaloid connections and their connectivity with the orbitofrontal gyrus (Yakovlev 1948). Yakovlev associated this circuit with emotions and motivations (Yakovlev 1948). Paul MacLean integrated the Papez and Yakovlev models as part of the limbic system and further modified and expanded the Papez circuit to include the prefrontal cortex, septum, and amygdalae (MacLean 1949, 1952). MacLean’s model further emphasized that the limbic cortex and the subcortical limbic structures were a functionally integrated system interconnected by both short and long-range fiber bundles (MacLean 1949, 1952).

The limbic system later extended to include the midbrain (Nauta and Domesick 1978; Nakano 1998). In 1982, Mishkin demonstrated that stimulation of the higher-order sensory areas of the cortex activates a cortico-limbo-thalamo-cortical circuit involved in monkeys’ memory function (Ungerleider et al. 1982). He proposed that this circuit may consist of two parallel loops, one involving the amygdala and the dorsomedial nucleus of the thalamus, and the other circuit may involve the hippocampus and the anterior nuclei such as the amygdala, septal and hypothalamic nuclei (Ungerleider et al. 1982). Some of the known limbic system functions in the human brain include learning, memory, olfaction, adjusting visceral functions, emotional processing, attention, and cognition (Nolte 2009).

Studies demonstrated direct engagement of the prefrontal cortex with the limbic system in primates, including the human brain (Robinson and Kolb 1997; Whalen et al. 1998; Barbas et al. 2003; Bryant et al. 2020; Rapan et al. 2021). The entire cerebral cortex is also indirectly connected to the anterior limbic nuclei such as the amygdalae, hypothalamic and septal nuclei mediated by the thalamus via extensive cortico-thalamic connectivity (Haines 2007).

In recent years, multiple additional limbic fiber connectivity has been revealed using diffusion-weighted imaging (DWI) techniques. The equivalent fiber connectivity of all these pathways has been documented by dissection studies in primates. Some of these fiber tracts include the amygdalofugal tract (AFT), amygdalothalamic tract (ATT), stria terminalis (ST), dorsal thalamo-hypothalamic tract (DTH), cerebello-hypothalamic tracts, and the parieto-occipito-hypothalamic tract (POHT) (Kamali et al. 2015; Kamali et al. 2016; Kamali et al. 2018a, b; Kamali et al. 2018a, b; Kamali et al. 2020a, b).

These newly described fiber tracts incorporate multiple additional limbic circuits to the already known complex limbic network. The purpose of this review is to assess the anatomy of the limbic structures and to elaborate on the anatomical connectivity of the limbic circuits as an update to the original Papez circuit. Please be advised that investigating possible functions of the limbic structures is beyond the scope and intent of this review. The acronyms used in the current review only imply the connectivity between the structures and do not imply any directionality to the neuronal impulse flow. For instance, the parieto-occipito-hypothalamic tract may transfer the neuronal impulse from the parieto-occipital cortices toward the hypothalamus, carry one-way information from the hypothalamus to the parieto-occipital lobes, or may be bidirectional. Please be advised that the words inside the parenthesis are the pathways connecting the structures in the upcoming circuits of this manuscript.

### The Papez circuit

The Papez circuit is composed of two parallel loops generated mainly by the cingulum bundle (CB) and fornix (FX) as follow:

$${\text{Prefrontal}}\,{\text{cingulate}}\,{\text{gyrus}}\, \Leftarrow \left( {{\text{CB}}} \right) \Rightarrow \,{\text{Parahippocampal}}\,{\text{gyrus}}\,\left( {{\text{temporal}}\,{\text{lobe}}} \right) \Leftarrow \left( {{\text{FX}}} \right) \Rightarrow {\text{Mammillary}}\,{\text{bodies}} \Leftarrow \left( {{\text{MTT}}} \right) \Rightarrow \,{\text{Ventral}}\,{\text{thalamus}} \Leftarrow \left( {{\text{TC}}} \right) \Rightarrow {\text{Prefrontal}}\,{\text{cingulate}}\,{\text{gyrus}}$$

**Fig. 1:**
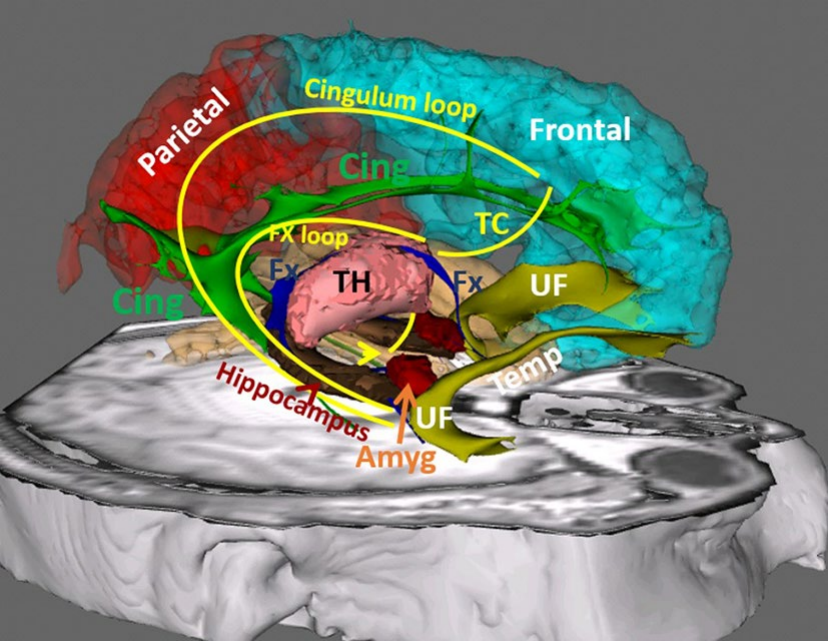
**A** 3-D reconstruction of the major gray and white matter structures of the limbic system including the Papez circuit. Frontal (cyan), parietal (red), and temporal (creamy color) cortices are shown in the background. Major gray matter nuclei of the limbic system are also shown, including the thalamus (TH in pink), hippocampus (brown), and amygdala (Amyg in red). Please note that the hypothalamus including the mammillary bodies is not shown, however, the mammillo-hypothalamic tract is shown by an arrowhead connecting the hypothalamus to the ventral thalamus. Major white matter pathways of the limbic system are shown, including the cingulum bundle (Cing in green), fornix (FX in blue), and uncinate fasciculus (UF in yellow). The Papez circuit is shown in yellow curved lines including the two parallel loops of cingulum loop and forniceal loop (FX loop). The two loops communicate with one another mediated by the thalamus via the mammillothalamic tract (shown by yellow arrowhead) and the thalamo-cortical fibers (TC). The Papez circuit consists of the frontoparietal connectivity with the parahippocampal gyrus/hippocampal formations via the cingulum bundle, hippocampal formations to mammillary bodies via the fornix, mammillary bodies to the thalamus via the mammillothalamic tract, and the thalamus to the frontoparietal lobes and cingulum bundle via the thalamocortical fibers (TC)

The CB is the longest limbic pathway of the Papez circuit generating a large loop connecting the prefrontal projections of the cingulate gyrus to the hippocampus in the parahippocampal gyrus of the temporal lobe (Fig. 1). The hippocampus then connects to the mammillary bodies via the fornix (FX). The mammillary bodies project to the ventral thalamus via the mammillothalamic tract (MTT). The ventral thalamus links back into the prefrontal cortex and anterior cingulate gyrus via the thalamo-cingulate projections (TC) to complete the Papez circuit (Fig. 1).

### The Yakovlev model/circuit

Yakovlev introduced the basolateral circuit and included the thalamo-amygdaloid connections and their connectivity with the orbitofrontal gyrus into the limbic system. Yakovlev associated the basolateral circuit with emotions (Yakovlev 1948). Orbitofrontal and cingulate gyri <=> temporal tip <=>  amygdala => mediodorsal thalamus <=> orbitofrontal and cingulate gyri

### The MacLean’s model

Paul MacLean integrated the Papez and Yakovlev models as part of the limbic system and further modified and expanded the Papez circuit to include the prefrontal cortex, septum, and amygdalae as part of the limbic system. This model has been accepted and almost unchanged ever since (MacLean 1949, 1952). MB = Mammillary bodies.
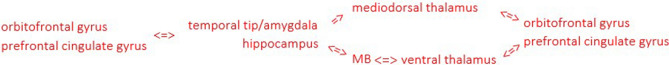

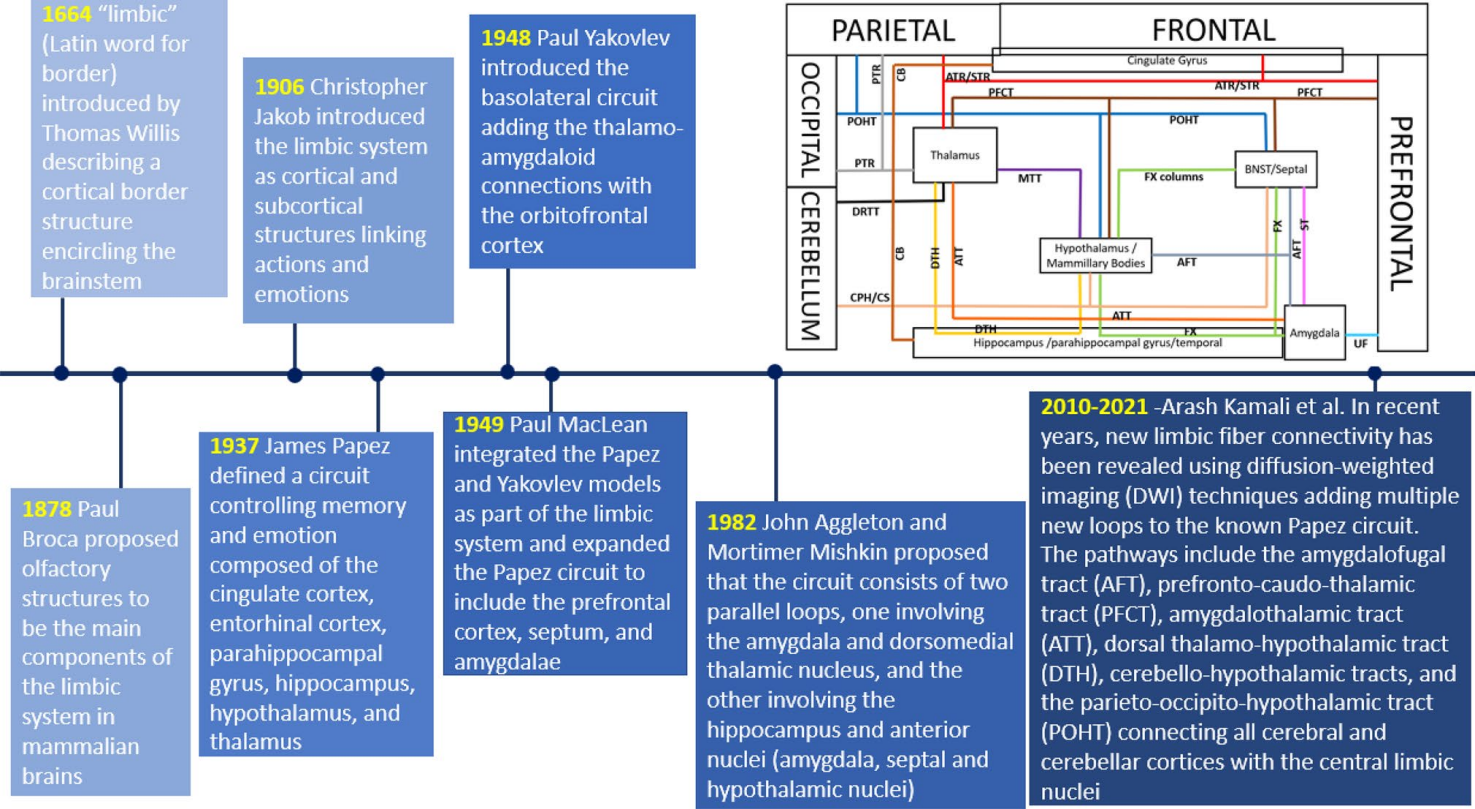


### The Cingulum Bundle (CB)

The cingulum, a Latin word for encircling structure, is the longest C-shaped limbic pathway which serves as the core of the Papez circuit by connecting prefrontal and parietal projections of the cingulate gyrus to the hippocampus in the parahippocampal gyrus of the temporal lobe (green fibers in Figs. 1, 3B). The cingulum bundle connects the anterior cingulate, dorsolateral and medial prefrontal, orbitofrontal cortices to the posterior cingulate, parietal, insular and parahippocampal, cortices and to the amygdala ventrally (Crosby 1963; Nieuwenhuys et al. 1988; Wakana et al. 2004; Schmahmann et al. 2007; Lawes et al. 2008). The CB is composed of a dorsal segment and a parahippocampal segment which are contiguous. The dorsal segment runs around the corpus callosum from the subgenual area and courses above the splenium of the corpus callosum alongside the superior longitudinal fasciculus I (Jang and Hong 2012; Kamali et al. 2014; Wang et al. 2016). The parahippocampal segment continues ventrally after a turn just posterior to the splenium of the corpus callosum, runs toward the anterior temporal lobe along the ventral aspect of the hippocampus and terminates in the temporal lobe (Catani et al. 2013; Pascalau et al. 2018).

### The Fornix (FX)

**Fig. 2:**
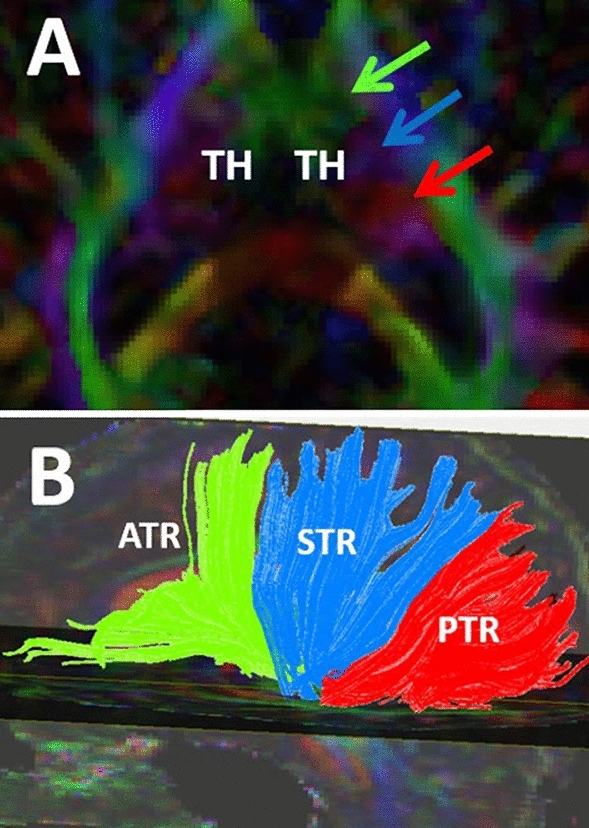
Axial (**A**) and 3-D (**B**) views of the high spatial resolution diffusion weighted imaging of brain parenchyma are shown. 2A shows the axial view of the thalami (TH) demonstrating three major colors. The anterior third of the thalamus contains the green fibers (green arrow), which run in anterior–posterior or posterior-anterior orientation, named the anterior thalamic radiations (ATR), shown as green fiber tracts in B. The ATR mostly project from the thalamus to the prefrontal cortex. The middle third of the thalamus contains the blue fibers (blue arrow), which are the cranio-caudally oriented fibers, named the superior thalamic radiations (STR). The STR is shown as blue projection fibers (**B**), projecting from the thalamus to the posterior frontal and parietal cortices (**B**). The posterior third of the thalamus includes the red fibers (red arrow) which course in latero-lateral direction, called the posterior thalamic radiations (PTR). The PTR fibers are shown in red (B) and are mostly projecting from the thalamus to the occipital cortex

**Fig. 3:**
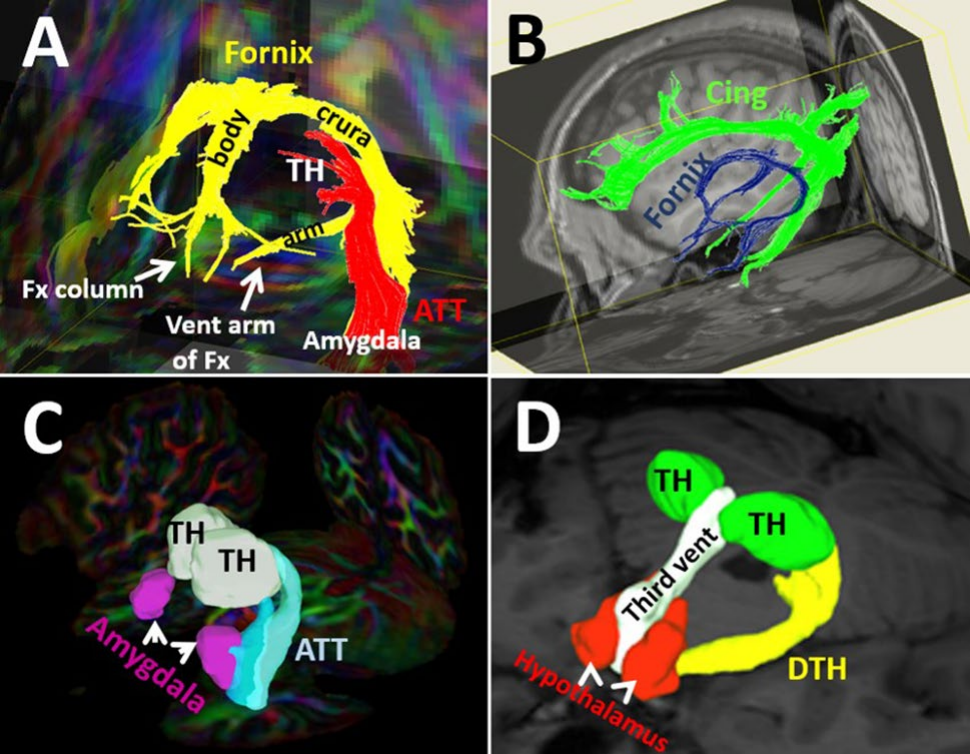
A-D represents the 3-D fiber tract reconstructions of the fornix, cingulum bundle, amygdalothalamic, and dorsal thalamo-hypothalamic tract. 3A. shows the relationship between the fornix (yellow) and the amygdalothalamic tract (ATT in red). Forniceal arms, crura, body, and columns are shown. 3B shows the relationship of the fornix (blue) and cingulum bundle (green). 3C illustrates the ATT (in cyan), arising from the dorsomedial thalamus and inserting into the amygdala. 3D illustrates the dorsal thalamo-hypothalamic tract (DTH in yellow) arising from the dorsomedial thalamus and inserting into the anterior hypothalamic nuclei. ATT, amygdalothalamic tract; Cing, cingulum bundle; DTH, dorsal thalamo-hypothalamic tract; Third vent, third ventricle; TH, thalamus

**Fig. 4:**
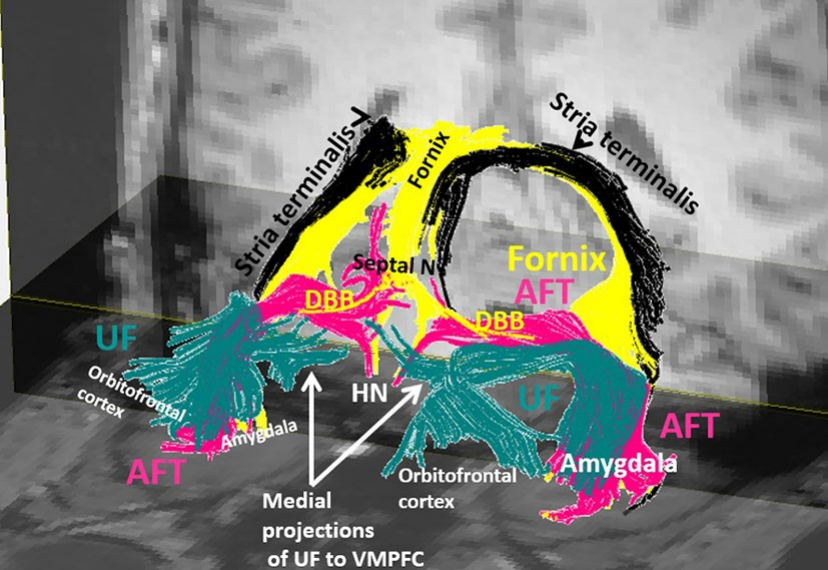
represents the 3-D fiber reconstruction of the fornix (yellow), stria terminalis (black), amygdalofugal tract (AFT in pink), and uncinate fasciculus (UF in green). The stria terminalis is shown connecting the amygdala to the region of septal nuclei (Septal N). The amygdalofugal tract is shown connecting the amygdala to the hypothalamic nuclei (HN) and septal nuclei (Septal N). The uncinate fasciculus is shown connecting the amygdala to the orbitofrontal cortex and ventromedial prefrontal cortex (VMPFC). The white arrows demonstrate the medial projections of the UF to the VMPFC. Diagonal band of Broca (DBB) are shown as the mustache like yellow fibers arising from the septal nuclei and course toward the Para olfactory regions bilaterally

The fornix is a long C-shaped fiber tract connecting the hippocampus and amygdala in the mesial temporal lobe to the hypothalamic and septal nuclei at the central base of the brain (Figs. 1, 2, 3A-B, 4).

The fornix connects the hippocampus to the mammillary body of the hypothalamus in the Papez circuit (Fig. 4). After exiting the hippocampus, the forniceal crura loops anteriorly alongside the lateral ventricles and form the forniceal body (Fig. 3A). The forniceal body is situated in the midline coursing anteriorly, and finally splits into forniceal columns at the front end. The forniceal columns course inferiorly and divide into the precommissural and postcommissural fibers (Figs. 3A, 4). The precommissural fibers traverse anteriorly to the anterior commissure, and the postcommissural fibers pass behind the anterior commissure. The precommissural fibers descend anterior to the lamina terminalis and end in the anterior septal nuclei. The postcommissural fibers take a C-shape course traversing through the posterior septal nuclei into the bed nucleus of the stria terminalis alongside the stria terminalis and continue inferiorly to reach and terminate in the hypothalamic nuclei (Fig. 4) (Lemaire et al. 2013; Kamali et al. 2015). Two ventral forniceal arms exit from the junction of the forniceal crura and fimbria away from the hippocampus coursing medially along the lateral margin of the cerebral peduncles toward the mammillary bodies and other hypothalamic nuclei (Fig. 3A) where they end (Lemaire et al. 2013; Kamali et al. 2015).

The fornix is the only limbic pathway that generates a complete loop on its own: a hypothalamo-septo-hippocampo-hypothalamic loop via the forniceal columns, body, crura, and ventral arms (Figs. 3A, 4).

$${\text{hypothalamic/septal}}\,{\text{nuclei}}\, \Leftarrow \,\left( {{\text{FX,}}\,{\text{body,}}\,{\text{crura}}} \right)\, \Rightarrow \,{\text{hippocampus}}\, \Leftarrow \,\left( {{\text{FX,}}\,{\text{ventral}}\,{\text{arms}}} \right)\, \Rightarrow \,{\text{hypothalamic}}/{\text{septal}}\,{\text{nuclei}}$$

### Diagonal Band of Broca (DBB)

The DBB fibers are horizontally oriented mustache-like fibers coursing very close to the precommissural fibers of the forniceal columns connecting the septal nuclei with the para olfactory and hippocampal regions (Fig. 4). The diagonal band of Broca are cholinergic fibers in the basal forebrain (Nolte 2009). This fiber tract may have a role as a direct connectivity between the septal nuclei and hippocampal/amygdala complex (Nolte 2009).

### The Mammillothalamic Tract (MTT)

The MTT is a small pathway serving as the primary route for the thalamo-hypothalamic connectivity in the Papez circuit (yellow arrow head in Fig. 1) (Yamada et al. 1998; Haines 2007). The hypothalamic nuclei, specifically the mammillary bodies, are involved in many limbic circuitry and directly or indirectly connected to the amygdala, hippocampus, and thalamus (Yamada et al. 1998; Kamali et al. 2018a, b). The mammillothalamic is a projection bundle that arises from the mammillary bodies, projects dorsally, and cranially, toward the thalamus. Along this course, the MTT ascends along the lateral aspect of the third ventricle to reach the anterior inferior aspect of the thalamus (Yamada et al. 1998). The MTT projects laterally within the anterior thalamus, and terminates in the anterior lateral thalamic nuclei (Yamada et al. 1998; Kamali et al. 2018a, b). The fornix and hippocampus are indirectly connected to the ventral thalamus via the mammillary bodies mediated by the MTT. Studies showed that the damage to the fornix and hippocampus indirectly results in damage to the ventral thalamus, likely by Wallerian degeneration through the MTT (Sutherland and Rodriguez 1989; Nolte 2009).

### The Hippocampus

The hippocampus is the largest gray matter nucleus of the limbic system residing in the mesial temporal lobe (Fig. 1) (Nolte 2009). The hippocampus is part of the original Papez circuit of the hippocampo-mammillo-thalamo-cingulate-hippocampal loop (Fig. 1). The hippocampus is connected to the anterior limbic nuclei, including the amygdala and hypothalamic/septal nuclei via multiple pathways. The hippocampus is connected to the hypothalamic nuclei via the ventral arms of the fornix. Moreover, the hippocampus is connected to the septal nuclei via the stria terminalis, forniceal fimbria, crura, and columns. The hippocampal head anatomically approximates and nearly contacts the amygdala (Nolte 2009) (Figss. 5, 6, 7, 8).

**Fig. 5:**
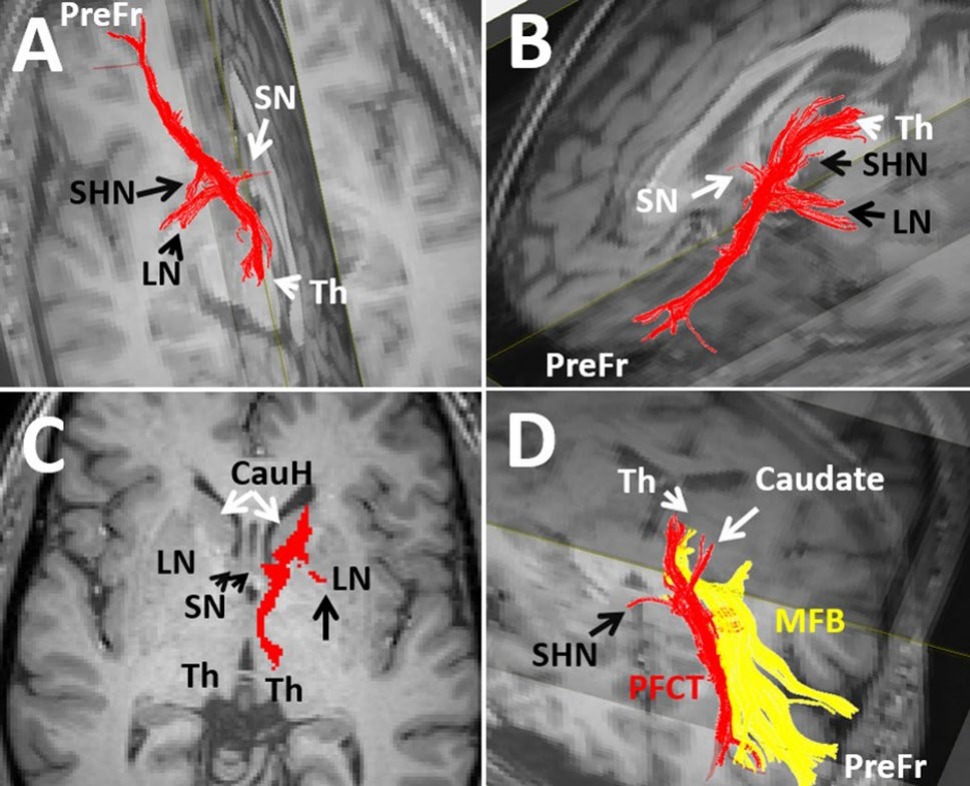
Different views of 3-D reconstructions of the prefronto-caudo-thalamic tract (PFCT in red) on T1 weighted backgrounds. 4A-D shows the connectivity of the PFCT with multiple gray matter nuclei, including the caudate head (CaudH), septal nuclei (SN), superior hypothalamic nuclei (SHN), lentiform nuclei (LN), and the thalamus (TH). The PFCT projects to the medial prefrontal cortex (PreFr). 4C shows the medial forebrain bundle (MFB in yellow), running side by side along the lateral and superior aspect of the PFCT inserting into the lateral prefrontal cortex (PreFr)

**Fig. 6:**
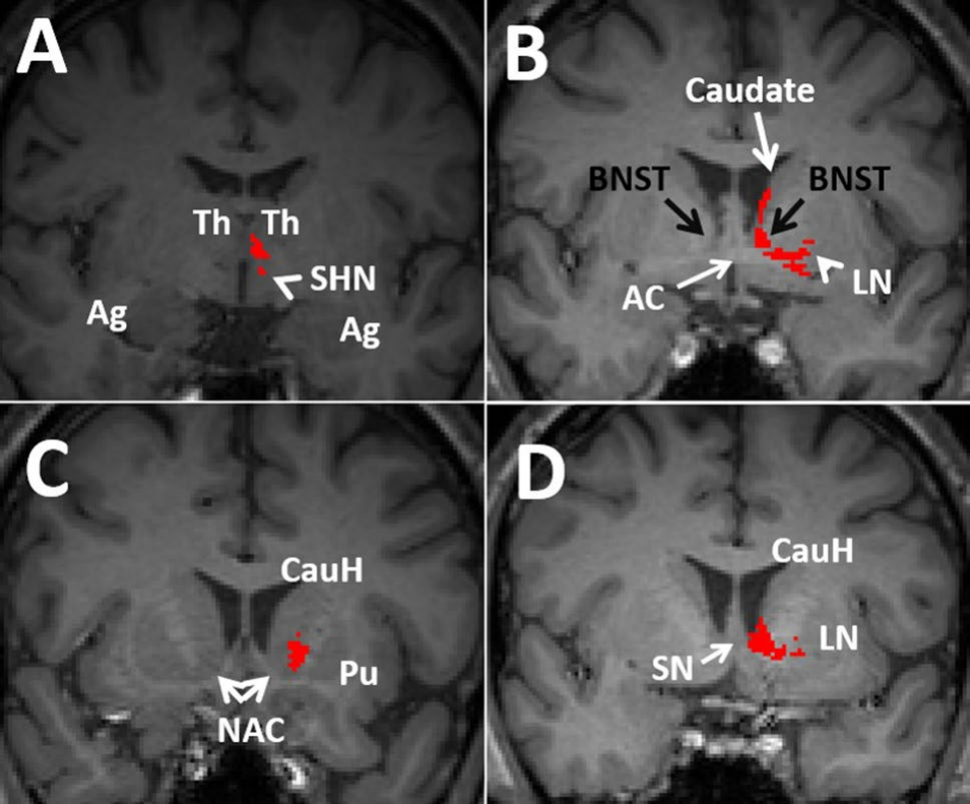
Four consecutive coronal T1 weighted anatomical views demonstrating the trajectory of the PFCT (red) from posterior (A) to anterior (D). The trajectory of the PFCT is shown arising from the dorsomedial thalamus (TH), projecting laterally and anteriorly along the lateral aspect of the third ventricle toward the caudate head (CauH, white arrow in C). The PFCT passes through the bed nucleus of stria terminalis (BNST), septal nuclei (SN), nucleus accumbens (NAC), and caudate head before projecting into the medial prefrontal cortex. Along the way, the PFCT receives projections from the superior hypothalamic nuclei (SHN, arrowhead in A) and lentiform nuclei (LN, arrowhead in B). Ag, amygdala; Pu, putamen; Th, thalamus

**Fig. 7:**
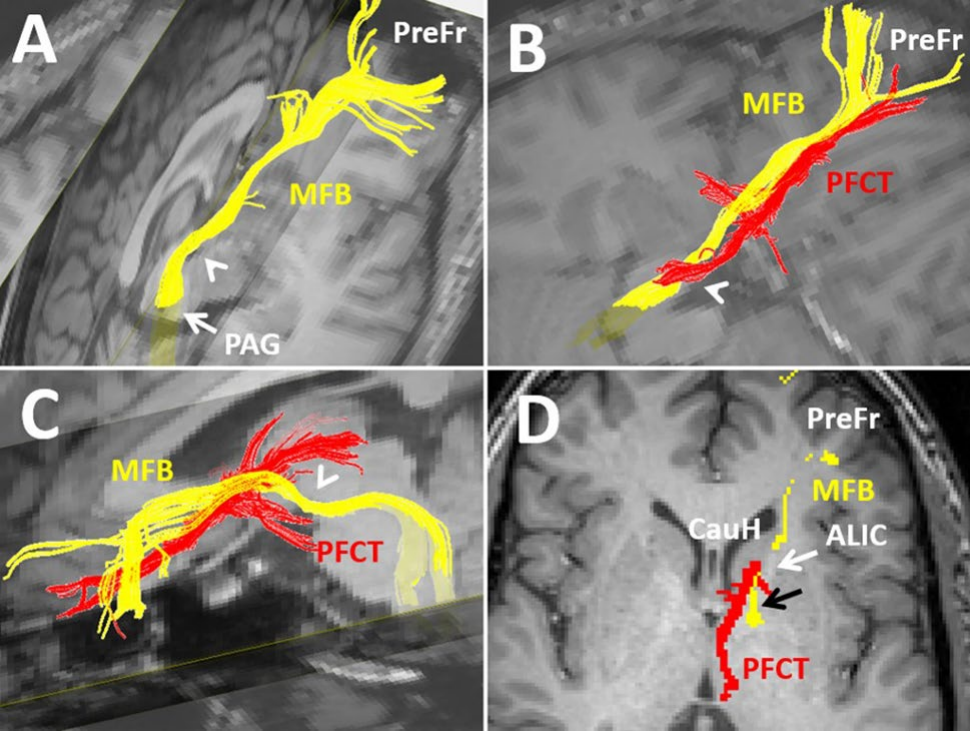
Different views of 3-D reconstructions of the prefronto-caudo-thalamic tract (PFCT in red) and the medial forebrain bundle (MFB in yellow) on T1 weighted imaging backgrounds. The MFB connects the midbrain's periaqueductal gray matter (PAG) to the ventral tegmental areas and the prefrontal cortex (PreFr). The MFB projects from the PAG anteriorly near the midline into the ventral tegmental area toward the interpeduncular cistern (marked by arrowhead in 7A-C). The MFB then courses superiorly toward the genu of the internal capsule (black arrow in 7D), where it enters the anterior limb of the internal capsule (ALIC). Unlike the MFB which courses through the ALIC into the prefrontal cortex, the PFCT courses outside the ALIC, more medially and through the caudate head before projecting to the medial prefrontal cortex (7B-D)

**Fig. 8:**
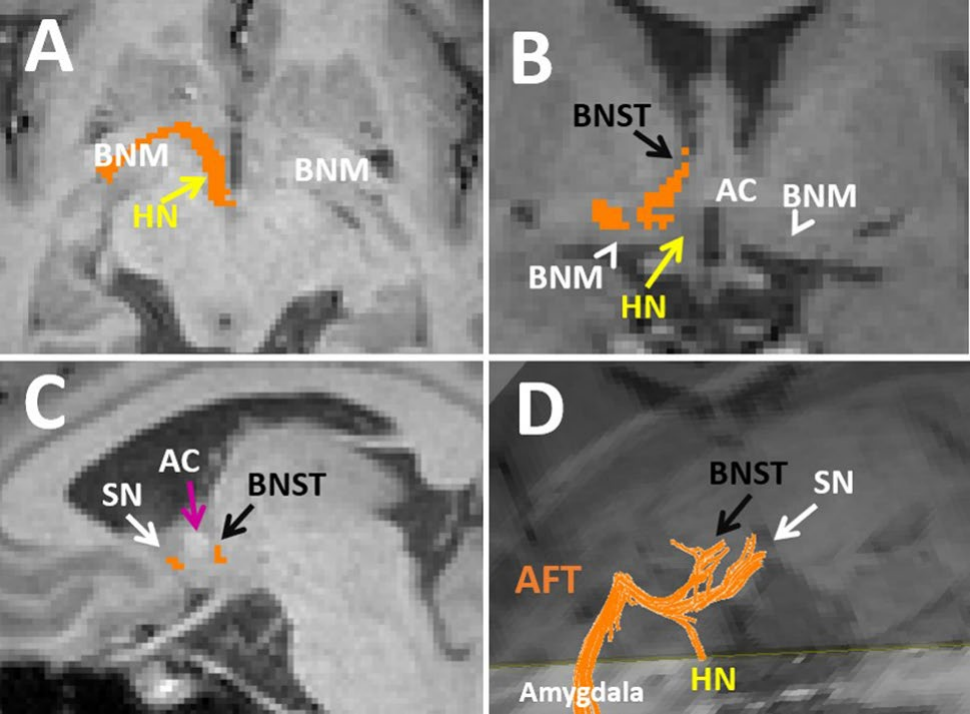
**A**-**C** trajectory of the amygdalofugal tract (orange) on T1 weighted backgrounds. 8D. 3-D reconstruction of the AFT is demonstrated. The AFT originates from the amygdala and projects superiorly and medially toward the midline. While projecting toward the midline (8B), the AFT courses through the basal nucleus of Meynert (BNM, arrowheads in 8B) along the inferior aspect of the anterior commissure (AC). The AFT splits into three groups of fibers near the midline (8D). Two groups of fibers project cranially toward the septal nuclei (SN, superior anterior marked by white arrows in 8C-D) and the bed nucleus of stria terminals (BNST, superior posterior marked by black arrows in 8B-D). The third group of fibers projects caudally into the hypothalamic nuclei (HN, yellow arrow in 8A) and ventral tegmental area

**Fig. 9:**
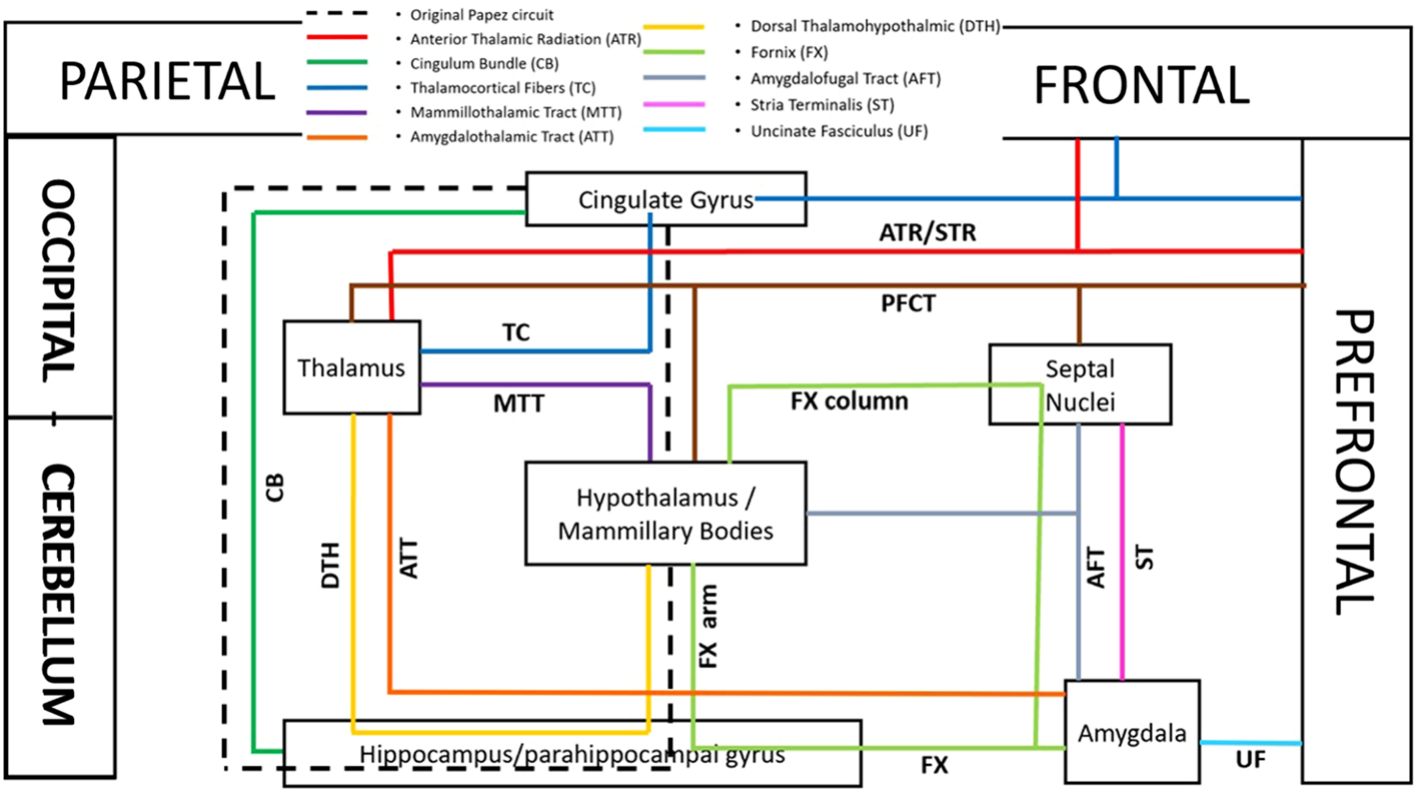
Schematic view of the major anterior neuronal circuits (fronto-temporal connectivity) of the limbic system mostly connected to the frontal and temporal cortices. The Papez circuit is shown by dotted box. The Papez circuit consists of the frontoparietal connectivity with the parahippocampal gyrus via the cingulum bundle, hippocampal formations to mammillary bodies via the fornix, mammillary bodies to the thalamus via the mammillothalamic tract (MTT), thalamus to the frontal lobe and cingulum bundle via the thalamocortical fibers (TC). By considering other limbic pathways such as the PFCT, ST, AFT, UF, ATT and DTH, several neuronal loops are added to the limbic system which were detailed in the text

The ventral forniceal arms unite the hippocampus with the hypothalamic nuclei including the mammillary bodies (Dillingham et al. 2015; Kamali et al. 2015, Dillingham et al. 2020). This connectivity along with the body and crura of the fornix contribute to a loop including the hypothalamic/septal nuclei (Fig. 9).

$${\text{hippocampus}}\, \Leftarrow \,\left( {{\text{FX,}}\,{\text{ventral}}\,{\text{arms}}} \right)\, \Rightarrow \,{\text{hypothalamus/septal}}\,{\text{nuclei}}\, \Leftarrow \left( {{\text{FX,}}\,{\text{body,}}\,{\text{crura}}} \right)\, \Rightarrow \,{\text{hippocampus}}$$

### The Thalamus

The human thalamus is at the center of the hippocampo-mammillo-thalamo-cingulate-hippocampal connectivity of the Papez circuit (Fig. 1). The thalamus has reciprocal connections with many structures including the cerebral cortex, basal ganglia, cerebellum, brainstem, and spinal cord (Lambert et al. 2017). The thalamus plays a critical role in modulating limbic functions through many connections with major bodies of the limbic system including the hippocampus, amygdala, hypothalamic and septal nuclei (Robertson and Kaitz 1981)(Fig. 1). In high spatial resolution diffusion-weighted imaging, the thalamus is divided into three segments by three major colors indicating different orientation of the thalamic fiber tracts (Fig. 2A). The anterior third of the thalamus is mostly green, indicating the anterior–posterior orientation of the anterior thalamic pathways such as the anterior thalamic radiations (ATR) (Fig. 2) (Aggleton et al. 2010; Kamali et al. 2010; Wilkinson et al. 2017; Grodd et al. 2020). The ATR fibers mostly project to the prefrontal cortex (Fig. 2A-B). The middle third of the thalamus shows up in blue color, which indicates the cranio-caudal orientation of the thalamic projections called the superior thalamic radiations (STR) (Fig. 2A-B). The STR consists of multiple pathways including the spinothalamic tract (Davidson et al. 2008, Kamali et al. 2009, Al-Chalabi and Gupta 2018). The posterior third of the thalamus is red in diffusion-weighted imaging, which indicates the latero-laterally oriented fiber tracts which are called the posterior thalamic radiations (PTR) (Fig. 2A-B). The PTR fibers mostly project to the occipital cortex and contain the optic radiations (Jellison et al. 2004; Sherbondy et al. 2008; Kamali et al. 2014; Nooij et al. 2015; Grodd et al. 2020).

$${\text{thalamus}}\, \Leftarrow \,\left( {\text{thalamo - cortical}} \right)\, \Rightarrow \,{\text{cerebral}}\,{\text{cortex}}\, \Leftarrow \,\left( {\text{cortico - thalamic}} \right) \Rightarrow \,{\text{thalamus}}$$

The thalamus is directly connected to vast areas of the cerebral cortex by many afferent and efferent projections including the anterior, superior and posterior thalamic radiations (ATR, STR and PTR). These include both reciprocal and non-reciprocal connections between the thalamus and cerebral cortex. The thalamocortical projections may project or receive neurons from the same cortical area or accept input from one cortical site and project to a different cortical area (Mandelbaum et al. 2019).

In addition to the ATR, the thalamus also links directly with the prefrontal cortex via the prefronto-caudo-thalamic tract (PFCT) (Kamali et al. 2010; Sun et al. 2018). The thalamus connects to the prefrontal cortex directly through the anterior limb of the internal capsule via the ATR or indirectly through the caudate nucleus and via the prefronto-caudo-thalamic tract (Kamali et al. 2010; Sun et al. 2018).

The thalamo-cortical radiations (ATR, STR, and PTR) and the PFCT play significant roles in many circuits involving the thalamus and vast areas of the cerebral cortices. Some of these circuits include the cortico-thalamo-limbo-cortical and cortico-thalamo-striata-cortical.

$${\text{cortex}}\, \Leftarrow \,\left( {\text{cortico - thalamic}} \right) \Rightarrow \,{\text{thalamus}}\, \Leftarrow \left( {{\text{thalamo}} - {\text{limbic}}} \right)\, \Rightarrow \,{\text{limbic}}\,{\text{nuclei}}\, \Leftarrow \,\left( {\text{limbo - cortical}} \right) \Rightarrow \,{\text{cortex}}$$

$${\text{cortex}}\, \Leftarrow \,\left( {\text{cortico - thalamic}} \right)\, \Rightarrow \,{\text{thalamus}}\, \Leftarrow \,\left( {{\text{thalamo}} - {\text{striatum}}} \right)\, \Rightarrow \,{\text{striatum}}\, \Leftarrow \,\left( {\text{striata - cortical}} \right)\, \Rightarrow \,{\text{cortex}}$$

$${\text{thalamus}}\, \Leftarrow \,\left( {\text{thalamo - striata}} \right)\, \Rightarrow \,{\text{striatum}}\, \Leftarrow \,\left( {\text{Striata - thalamic}} \right)\, \Rightarrow \,{\text{thalamus}}$$

The thalamus also directly connects with multiple limbic system nuclei, including the hypothalamic/septal nuclei, hippocampus, and amygdala. The mammillothalamic tract (MTT) connects the ventral thalamus to the mammillary bodies of the hypothalamus (Otake et al. 1994; Aggleton et al. 2010; Kamali et al. 2018a, b; Grodd et al. 2020). The dorsal thalamo-hypothalamic (DTH) tract is another connectivity between the dorsal thalamus and anterior hypothalamus. The DTH connects the pulvinar, lateroposterior (LP) nucleus and midline nuclei of the thalamus to the hippocampus and to the ventral hypothalamic nuclei (Fama and Sullivan 2015; Kamali et al. 2020a, b). The thalamus is also directly connected to the amygdala via the amygdalothalamic tract (ATT) (Otake et al. 1994; Aggleton et al. 2010; Kamali et al. 2018a, b; Grodd et al. 2020). These pathways create multiple circuits involving the thalamus as follows (Fig. 9).

$${\text{thalamus}}\, \Leftarrow \,\left( {{\text{DTH}}} \right)\, \Rightarrow \,{\text{hippocampus}}\, \Leftarrow \,\left( {{\text{DTH}}} \right)\, \Rightarrow \,{\text{hypothalamus}}\, \Leftarrow \,\left( {{\text{MTT}}} \right)\, \Rightarrow \,{\text{thalamus}}$$

$${\text{thalamus}}\, \Leftarrow \,\left( {{\text{DTH}}} \right)\, \Rightarrow \,{\text{hippocampus}}\, \Leftarrow \,\left( {{\text{DTH}}} \right)\, \Rightarrow \,{\text{hypothalamus}}\, \Leftarrow \,\left( {{\text{FX}}} \right)\, \Rightarrow \,{\text{amygdala}}\, \Rightarrow \,\left( {{\text{ATT}}} \right)\, \Rightarrow \,{\text{thalamus}}$$

### The Anterior Thalamic Radiations (ATR)

The anterior thalamic radiation (ATR) mainly connects the thalamus to the prefrontal cortices and contains the afferent and efferent projections from the anterior thalamus to the prefrontal cortex. The ATR is composed of reciprocal fibers that run between the thalamus and prefrontal cortex along the most medial aspect of the anterior limbs of the internal capsules (Kahle et al. 2002, Zhou et al. 2003; Kamali et al. 2010; Mamah et al. 2010). More specifically, the ATR fibers connect the thalamic midline and anterior nuclear groups with the frontal lobe by traversing through the anterior limbs of bilateral internal capsules (George and Das 2020) (Fig. 2B). The ATR and medial forebrain bundle (MFB) are both major fiber projections projecting through the anterior limb of the internal capsule toward the prefrontal cortex. The ATR runs medial to the medial forebrain bundle in the anterior limb of the internal capsule (Coenen et al. 2012; Pascalau et al. 2018).

### The Superior Thalamic Radiations (STR)

The STR is a connection between the ventral nuclear group of the thalamus and the pre and post-central gyrus (Fig. 2B) (George and Das 2020). The superior thalamic radiation (STR) includes the afferent and efferent fibers connecting the thalamus with the posterior frontal and parietal cortices. For instance, the STR includes the thalamo-cortical projections of the spinothalamic tract to the parietal cortex which is the major somatosensory fiber system of the human body. The STR also consists of the efferent projections contiguous with the medial lemniscus into the frontoparietal cortices (Kamali et al. 2009), which is also part of the somatosensory fiber system. Further, the STR includes the thalamo-cortical projections of the dentato-rubro-thalamic tract, (DRTT) projecting from the thalamus to the motor and premotor cortical areas of the frontal lobe. The DRTT is the major cerebellar output pathway, which carries the motor information from the cerebellum to the motor cortex via the brainstem and thalamus (Benagiano et al. 2018, Keser et al. 2021).

### The Posterior Thalamic Radiations (PTR)

The PTR serves as a connection between the occipital and parietal lobes and the thalamus by passing through the retro lenticular limb of the internal capsule (Fig. 2B) (George and Das 2020). The thalamus ties into broad areas of the parieto-occipital cortices through the posterior thalamic radiation (PTR), including the afferent and efferent fibers connecting the posterior thalamic nuclei to the parieto-occipital cortices and includes the optic radiations (Kamali et al. 2010, Nooij et al. 2015].

### The Thalamo-Cingulate Connections (TC)

The thalamo-cingulate connections (TC) are the fibers arising from the thalamic nuclei and project into the cingulum bundle to complete the Papez circuit mediating the thalamo-cingulate connectivity (Jang and Yeo 2013, 2014). The TC fibers maybe a component of the thalamo-cortical fibers of the ATR bundle.

The TC serves as a bidirectional and direct pathway between the thalamic nuclei, ascends through the internal capsule, and reaches the cingulate gyrus (Jones 2012; Jang and Yeo 2013).

### The Dorsal Thalamo-Hypothalamic Tract (DTH)

In addition to the MTT, which serves as the major ventral thalamo-hypothalamic pathway, the dorsal thalamo-hypothalamic tract (DTH) was recently described in the human brain as a dorsal thalamo-hypothalamic connectivity (Fig. 3D) (Kamali, et al. 2020a). The DTH connects the dorsal thalamus to the hippocampus and into the anterior hypothalamic nuclei. The DTH is a much larger pathway in volume compared to the MTT. Unlike the MTT, which connects the dorsal hypothalamus (mammillary bodies) to the anterior thalamus, the DTH connects the dorsal thalamus to the anterior hypothalamus (Fig. 3D) (Aggleton et al. 2010; Peterson et al. 2019; Kamali et al. 2020a, b). Several animal studies have revealed an equivalent fiber tract connecting the thalamus to the hippocampus and hypothalamus (Conrad and Pfaff 1976; Pasquier and Reinoso-Suarez 1976; Swanson 1976; Herkenham 1978; Cornwall and Phillipson 1988; Groenewegen 1988; Chen and Su 1990; Krout and Loewy 2000).

Aggleton and Mishkin proposed a dual circuit for memory in monkeys (Aggleton and Mishkin 1982). One circuit involving the amygdala and the dorsomedial nucleus of the thalamus, and a second circuit connecting the hippocampus to the anterior limbic nuclei, including the amygdala and hypothalamus. The DTH, along with the fornix/stria terminalis, may play a part in the second circuit and the thalamo-hippocampo-hypothalamic pathway proposed by Aggleton and Mishkin (Aggleton and Mishkin 1982). The DTH adds a parallel route to the thalamo-hippocampo-hypothalamic connectivity alongside the Papez circuit by directly connecting the thalamus to the hippocampus and hypothalamic nuclei which previously known to be feasible via the fornix (hippocampo-hypothalamic) and MTT (hypothalamo-thalamic) in the Papez circuit. Therefore, the DTH may contribute to many limbic circuits alongside the Papez circuit. The DTH and the MTT together may generate a thalamo-hippocampo-hypothalamo-thalamic circuit (Fig. 9).

$${\text{thalamus}}\, \Leftarrow \,\left( {{\text{DTH}}} \right)\, \Rightarrow \,{\text{hippocampus}}\, \Leftarrow \,\left( {{\text{DTH,}}\,{\text{FX}}} \right)\, \Rightarrow \,{\text{hypothalamus}}\, \Leftarrow \,\left( {{\text{MTT}}} \right)\, \Rightarrow \,{\text{thalamus}}$$

### The Amygdalothalamic Tract (ATT)

The ATT is the largest direct amygdalo-diencephalic connection in primates providing a direct connectivity between the amygdala and the dorsomedial nucleus of the thalamus (Fig. 3A,C) (Kamali et al. 2018a, b). The ATT serves as the amygdalothalamic limb of the ventral amygdalofugal complex, which includes the amygdalo-hypothalamic, amygdalo-septal, amygdalo-mesencephalic, and amygdalo-thalamic tracts (Noback et al. 2005, Haines 2007). The amygdala projects directly to the orbitofrontal cortex via the uncinate fasciculus. However, the amygdala also directly connects with the dorsomedial nucleus of the thalamus via the ATT (Fig. 9) (Noback et al. 2005, Haines 2007) and indirectly to the prefrontal cortex via the ATR mediated by the thalamus (Fig. 9).

Aggleton and Mishkin proposed a dual circuit for memory in monkeys (Aggleton and Mishkin 1982). Mishkin demonstrated that stimulation of the higher-order sensory areas of the cortex activates a cortico-limbo-thalamo-cortical circuit. This circuit may actually consist of two parallel circuits, one involving the amygdala and the dorsomedial nucleus of the thalamus and the other the hippocampus and the anterior nuclei (Aggleton and Mishkin 1982). The circuit involving the amygdala and the dorsomedial nucleus of the thalamus is likely via the amygdalothalamic tract, connecting the amygdala to the dorsomedial nucleus of the thalamus (Kamali et al. 2018a, b).

Some of the fibers of the ATT continue beyond the amygdala into the anterior temporal lobe (Klingler and Gloor 1960; Kiernan 2012; Kamali et al. 2018a, b). Additional small projections are also described between the anterior temporal pole and basomedial and basolateral nuclei of the amygdala through a small white matter tract called the “amygdalo-temporal fascicle” (Klingler and Gloor 1960; Ghashghaei and Barbas 2002).

The ATT may participate in multiple circuits of the limbic system, some of which are listed below (Fig. 9).

$${\text{amygdala}}\, \Rightarrow \,\left( {{\text{ATT}}} \right)\, \Rightarrow \,{\text{thalmaus}}\, \Leftarrow \,\left( {\text{DTH/MTT}} \right)\, \Rightarrow \,{\text{hypothalamus}}\, \Leftarrow \,\left( {\text{FX/ST}} \right)\, \Rightarrow \,{\text{hippocampus/amygdala}}$$

$${\text{orbitofrontal/prefrontal}}\,{\text{cortex}}\, \Leftarrow \,\left( {{\text{UF,}}\,{\text{CB}}} \right)\, \Rightarrow \,{\text{temporal}}\,{\text{tip}}\, \Leftarrow \,\left( {{\text{UF}}} \right)\, \Rightarrow \,{\text{amygdala}}\, \Rightarrow \,\left( {{\text{ATT}}} \right)\, \Rightarrow \,{\text{mediodorsal}}\,{\text{thalamus}}\, \Leftarrow \,\left( {\text{ATR/PFCT}} \right)\, \Rightarrow \,{\text{orbitofrontal/prefrontal}}\,{\text{cortex}}$$

$${\text{amygdala}}\, \Rightarrow \,\left( {{\text{ATT}}} \right)\, \Rightarrow \,{\text{thalmus}}\, \Leftarrow \,\left( {{\text{ATR}}} \right)\, \Rightarrow \,{\text{prefrontal}}\,{\text{cortex}}\, \Leftarrow \,\left( {{\text{UF}}} \right)\, \Rightarrow \,{\text{amygdala}}$$

The ATT is also part of the Yakovlev model of the basolateral circuit as follow:

$${\text{Yakovlev model:}}\,{\text{orbitofrontal}}\,{\text{and}}\,{\text{cingulate}}\,{\text{gyri}}\, \Leftarrow \,\left( {\text{UF, CB}} \right)\, \Rightarrow \,{\text{temporal}}\,{\text{tip/amygdala}}\, \Rightarrow \,\left( {{\text{ATT}}} \right)\, \Rightarrow \,{\text{mediodorsal}}\,{\text{thalamus}}\, \Leftarrow \,\left( {{\text{ATR}}} \right)\, \Rightarrow \,{\text{orbitofrontal}}\,{\text{and}}\,{\text{cingulate}}\,{\text{gyri}}$$

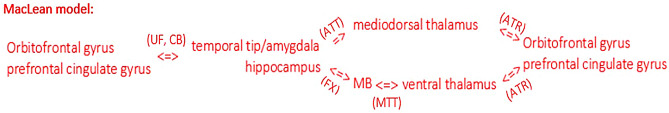


### The Prefronto-Caudo-Thalamic Tract (PFCT)

This pathway was first described in 2010 (Kamali et al. 2010). The PFCT is a large projection bundle connecting the thalamus to the basal ganglia, includes the caudate nucleus and lentiform nuclei and eventually projects into the prefrontal cortex (Figs. 5–7). The PFCT contains some of the cortico-striatal pathways between the putamina and caudate nuclei to the prefrontal cortex (Fig. 5C). The PFCT directly connects the thalamus with the BNST and septal nuclei (Figs. 5C, 6B). The PFCT communicates with multiple limbic nuclei, including the hypothalamic, septal, BNST, and nucleus accumbens. The ATR and PFCT both project from the thalamus to the prefrontal cortex. The ATR traverses through the anterior limb of the internal capsule, while the PFCT traverses more medially through the caudate nucleus to reach the prefrontal cortex (Kamali et al. 2010).

At the thalamic origin, the PFCT has a segment with convexity toward the midline and concavity toward the lateral thalamus, which arises from the dorsomedial (DM) nucleus of the thalamus. After arising from the DM nucleus of the thalamus, the PFCT projects into the BNST and receives projections from the septal nuclei and lentiform nuclei (Fig. 5–6). Aside from the septal nuclei sending projections to the PFCT medially, there are other projections along the way joining the PFCT. These projections are joining the PFCT superiorly from the caudate body (Fig. 6B), laterally from the lenticular nuclei (putamen and globus pallidus) (Figs. 5C, 6B), and inferiorly from the hypothalamic nuclei (Fig. 6A). The PFCT connects all these structures with the caudate head, nucleus accumbens, and prefrontal cortex.

The PFCT then courses through the caudate head and nucleus accumbens (Fig. 6C) and eventually projects into the medial prefrontal cortex (Haber et al. 1995; Kamali et al. 2010). The trajectories of the PFCT and medial forebrain bundle (MFB) are shown in Fig. 7 for better orientation of the readers to the courses of these tracts. The ATR, similar to the MFB, courses through the anterior limb of the internal capsule (ALIC). The PFCT connects the thalamus, basal ganglia, and multiple additional gray matter nuclei to one another and eventually to the prefrontal cortex. Therefore, the PFCT may serve as thalamo-prefrontal, hypothalamo-prefrontal, septo-prefrontal, and BNST-prefrontal conduits within the cortico-thalamo-limbo-cortical circuit. By means of connectivity, the PFCT may also be a part of many limbic circuits, including the prefronto-thalamic, prefronto-hypothalamic, prefronto-septal, and prefronto-striatal (Fig. 9).

Some of the circuits involving the PFCT are detailed below (Fig. 9).

$${\text{orbitofrontal/prefrontal}}\,{\text{cortex}}\, \Leftarrow \,\left( {{\text{UF,}}\,{\text{CB}}} \right)\, \Rightarrow \,{\text{amygdala}}\, \Rightarrow \,\left( {{\text{ATT}}} \right)\, \Rightarrow \,{\text{mediodorsal}}\,{\text{thalamus}}\, \Leftarrow \,\left( {{\text{PFCT}}} \right)\, \Rightarrow \,{\text{caudate/nuc}}\,{\text{accumbens}}\, \Leftarrow \,\left( {{\text{PFCT}}} \right)\, \Rightarrow \,{\text{orbitofrontal/prefrontal}}\,{\text{cortex}}$$

$${\text{prefrontal}}\,{\text{cortex}}\, \Leftarrow \,\left( {\text{PFCT/ATR}} \right)\, \Rightarrow \,{\text{thalamus}}\, \Leftarrow \,\left( {{\text{DTH}}} \right)\, \Rightarrow \,{\text{hypothalamus}}\, \Rightarrow \,\left( {{\text{PFCT}}} \right)\, \Rightarrow \,{\text{prefrontal}}\,{\text{cortex}}$$

$${\text{prefrontal}}\,{\text{cortex}}\, \Leftarrow \,\left( {{\text{PFCT}}} \right)\Rightarrow\,{\text{ventral}}\,{\text{striatum}}\, \Leftarrow \,\left( {{\text{mesolimbic}}\,{\text{pathway}}} \right)\, \Rightarrow \,{\text{ventral}}\,{\text{tegmental}}\,{\text{area}}\, \Leftarrow \,\left( {{\text{MFB}}} \right)\, \Rightarrow \,{\text{prefrontal}}\,{\text{cortex}}$$

### The Amygdala

The amygdala, a Latin word for almond, is an almond-shaped cluster of deep gray matter nuclei located bilaterally, just anterior to the hippocampus in the medial temporal lobe, (Figs. 1, 2C) and is the most ventrally located limbic nucleus. Due to massive communication with multiple limbic and non-limbic brain structures, it is not surprising that the amygdala is shown to control many cognitive and behavioral functions such as fear, aggression, anxiety, associative learning, attention, memory, social interaction, sexual orientation, decision-making, visual processing, processing emotions and facial emotions (Gallagher and Holland 1994; Davis and Whalen 2001; Walf and Frye 2006; Bocchio et al. 2016; Zinn et al. 2016; Haller 2018; Ressler and Maren 2019, Rudzinskas et al. 2019).

The anterior connectivity of the amygdala with the orbitofrontal cortex and the anterior temporal lobes is via the uncinate fasciculus (UF) (Fig. 4) (Di et al. 2017). The amygdala is in contact with the temporal and occipital lobes via the inferior longitudinal fasciculus (Di et al. 2017) and via additional direct fiber connectivity with parietal and occipital cortices (Nguyen et al. 2016). In a recent study, we hinted to the possibility of a direct posterior connectivity of the amygdala with the parieto-occipital cortices (Kamali et al. 2020b). The amygdala is also in direct connectivity with the brainstem via the amygdalofugal tract (AFT) (Noback et al. 2005, Nolte 2009). It is worth noting that all of these connections could be bi-hemispheric, since the bilateral amygdalae are interconnected to one another via the anterior commissure.

The amygdala is directly connected to the cingulate gyrus and wide areas of cerebral cortex via the cingulum bundle (CB). The amygdala is also indirectly connected to wide areas of the cerebral cortex through the thalamus via the amygdalothalamic tract (ATT). The amygdala is in connection with the prefrontal cortex indirectly through the hypothalamic/septal nuclei via the amygdalofugal tract (AFT) (connecting the amygdala to the hypothalamic/septal nuclei) and PFCT (connecting the hypothalamic/septal nuclei to the prefrontal cortex) (Fig. 9).

The amygdala is a major element of the amygdalo-thalamo-prefronto-amygdala circuit. The amygdalo-thalamic route via ATT, thalamo-prefrontal via ATR and PFCT, and prefronto-amygdala route via the uncinate fasciculus (UF) and cingulum bundle (Fig. 9).

$${\text{amygdala}}\, \Rightarrow \,\left( {{\text{ATT}}} \right)\, \Rightarrow \,{\text{thalamus}}\, \Leftarrow \,\left( {{\text{ATR}}} \right)\, \Rightarrow \,{\text{prefrontal}}\,{\text{cortex}}\, \Leftarrow \,\left( {{\text{UF,}}\,{\text{CB}}} \right)\, \Rightarrow \,{\text{amygdala}}$$

The amygdala is a part of the amygdalo-prefronto-septo-hypothalamo-hippocampo-amygdala loop via the UF and CB (amygdalo-prefrontal), PFCT (prefronto-septal/hypothalamic), and fornix (septal/hypothalamic-hippocampo-amygdala).

$${\text{amygdala}}\, \Leftarrow \,\left( {{\text{UF,}}\,{\text{CB}}} \right)\, \Rightarrow \,{\text{prefrontal}}\,{\text{cortex}}\, \Leftarrow \,\left( {{\text{PFCT}}} \right)\, \Rightarrow \,{\text{hypothalamus/septal}}\, \Leftarrow \,\left( {{\text{FX}}} \right)\, \Rightarrow \,{\text{hippocampus}}\, \Leftrightarrow \,{\text{amygdala}}$$

### The Uncinate Fasciculus (UF)

The UF is a bidirectional, U-shaped direct and monosynaptic cortico-cortical bundle that reciprocally connects the inferior frontal gyrus, orbitofrontal cortex, and inferior surfaces of the frontal lobe, including the ventromedial prefrontal cortex to the anterior temporal lobe (Fig. 1,4) (Ebeling and Cramon 1992, Ghashghaei and Barbas 2002, Kier et al. 2004, Schmahmann et al. 2008, Von Der Heide et al. 2013).

The UF is the most laterally located limbic white matter pathway. The UF’s lateral side curves upward into the extreme and external capsule and medial to the insular cortex. It has connections with the lenticular nucleus, internal capsule, and medial insular cortex, and then continues into the orbitofrontal cortex (Kier et al. 2004; Pascalau et al. 2018). Additional medial projections of the UF terminate in the ventromedial prefrontal cortex, including the para olfactory areas (Fig. 4) (Whalen et al. 1998).

The UF participates in the cortico-limbo-cortical loop. The UF is a part of the prefronto-amygdalo-hypothalamo-septo-caudate-prefrontal loop where the prefronto-caudo-septo/hypothalamic connectivity is run by the PFCT, the thalamo-amygdaloid connectivity executed by the ATT and the amygdalo-prefrontal connectivity is via the UF (Fig. 9).

$${\text{orbitofrontal/prefrontal}}\,{\text{cortex}}\, \Leftarrow \,\left( {{\text{UF}}} \right)\, \Rightarrow \,{\text{temporal}}\,{\text{tip}}\, \Leftarrow \,\left( {{\text{UF}}} \right)\, \Rightarrow \,{\text{amygdala}}\, \Rightarrow \,\left( {{\text{ATT}}} \right)\, \Rightarrow \,{\text{mediodorsal}}\,{\text{thalamus}}\, \Leftarrow \,\left( {\text{ATR/PFCT}} \right)\, \Rightarrow \,{\text{orbitofrontal/prefrontal}}\,{\text{cortex}}$$

### The Ventral Amygdalofugal Tract (AFT)

The ventral amygdalofugal tract consists of afferent and efferent connections to the basal forebrain, hypothalamus, and thalamus (Mori et al. 2017). The ventral amygdalofugal complex consists of the most crucial amygdalo-diencephalic connections in the human brain, including the amygdalo-hypothalamic, amygdalo-septal, amygdalo-thalamic, and amygdalo-mesencephalic tracts (Fig. 4, 8) (Noback et al. 2005, Kamali et al. 2016). The AFT plays a pivotal role in central limbic connections and allows the amygdala to control the hypothalamic/septal nuclei and BNST via the amygdalo-hypothalamic, amygdalo-septal, and amygdalo-BNST connections (Noback et al. 2005, Nolte 2009; Usunoff et al. 2009; Kamali et al. 2015).

The amygdalo-hypothalamic and amygdalo-septal components of the AFT are hook-like structures in the basal forebrain running parallel along the posterior-inferior aspect of the anterior commissure (Fig. 8) (Kamali et al. 2016). In the midline, some of the superior projections of the AFT project toward and terminate at the BNST, providing a direct amygdalo-BNST connectivity (Weller and Smith 1982).

Unlike the anterior commissure, which is continuous in the midline connecting the amygdalae to one another, the AFT runs just posterior and parallel to the anterior commissure. The AFT originates from the basolateral nucleus and central nucleus of the amygdala and projects posteriorly and superiorly toward the striatum (Noback et al. 2005, Nolte 2009). The AFT then turns medially and enters the gray matter structure of the basal nucleus of Meynert before reaching the midline (Fig. 8A, C). The AFT continues through the nucleus basalis toward the hypothalamic nuclei. Near the midline, the AFT splits into three groups of fibers. Some of the fibers course cranially and anteriorly to terminate at the septal nuclei (Fig. 8C). The second group of cranial fibers courses cranially and slightly posteriorly into the BNST (Fig. 8B-D). A third group of fibers splits near the midline and courses caudally into the hypothalamic nuclei and ventral tegmentum (Fig. 8A). Some of the circuits involving the AFT are listed below.

$${\text{amygdala}}\, \Rightarrow \,\left( {{\text{AFT}}} \right)\, \Rightarrow \,{\text{hypothalamus/septal}}\,{\text{nuc}}\, \Leftarrow \,\left( {\text{PFCT/MFB}} \right)\, \Rightarrow \,{\text{prefrontal}}\,{\text{cortex}}\, \Leftarrow \,\left( {{\text{UF,}}\,{\text{CB}}} \right)\, \Rightarrow \,{\text{amygdala}}$$

$${\text{orbitofrontal/prefrontal}}\,{\text{cortex}}\, \Leftarrow \,\left( {{\text{UF,}}\,{\text{CB}}} \right)\, \Rightarrow \,{\text{amygdala}}\, \Rightarrow \,\left( {{\text{AFT}}} \right)\, \Rightarrow \,{\text{hypothalamic/septal}}\, \Leftarrow \,\left( {{\text{PFCT}}} \right)\, \Rightarrow \,{\text{caudate/nuc}}\,{\text{accubmbens}}\, \Leftarrow \,\left( {{\text{PFCT}}} \right)\, \Rightarrow \,{\text{orbitofrontal/prefrontal}}\,{\text{cortex}}$$

### The Stria Terminalis (ST)

The ST is a major amygdalo-septal/hypothalamic connectivity of the limbic system and is believed to be one of the major output pathways of the amygdala (Nolte 2009). The stria terminals and fornix are the two major pathways connecting the hippocampus with all three major anterior limbic nuclei, including the amygdala, hypothalamic, and septal nuclei. The ST also has connectivity with the bed nucleus of stria terminalis (BNST). The ST courses side by side with the fornix along the medial margin of the lateral ventricles. The ST follows a C-shaped course laterally and parallel to the fornix, descends through the caudothalamic groove, and reaches the hypothalamic and septal nuclei and the bed nucleus of the ST (Noback et al. 2005, Lövblad et al. 2014, Kamali et al. 2015).

### The Anterior Commissure (AC)

The AC is a fiber bundle in front of the columns of the fornix, which connects structures in the right and left temporal lobes, including amygdalae, across the midline. In other words, as part of the commissural pathways, the AC, along with the corpus callosum, posterior commissure, and hippocampal commissure, allow for communication between the cerebral hemispheres. The AC is believed to play an essential role in multiple limbic circuits by connecting the amygdalae to one another. For instance, the AC operates as the amygdalo-amygdala limb of the amygdalo-thalamo-amygdalo-amygdala loop connecting the amygdalae to one another. The AC is believed to be involved in pain sensation by relaying information between the two amygdalae. The AC may also play a role in emotional regulation and memory.

### The Hypothalamus (Hypothalamic Nuclei)

The hypothalamus is a cluster of anterior diencephalic nuclei which is at the center of the limbic system anatomically. The hypothalamus is a collection of nuclei located lateral to the third ventricle and superior and posterior to the optic chiasm (Fig. 3D). Similar to the amygdala, the hypothalamic and septal nuclei are among the most connected structures of the limbic system. The hypothalamus is directly connected to wide areas of the cerebral cortex, including the frontal, parietal, occipital, and temporal lobes, along with the cerebellum and mesencephalon. A recent high-resolution diffusion-weighted tractography study showed direct parieto-occipital cortical connectivity with hypothalamic nuclei in the human brain (Kamali et al. 2020b), which may be involved in the transfer of the visuosensory information to the hypothalamic nuclei. The hypothalamus is also connected with the cerebellar hemispheres via the direct (cerebello-hypothalamic) and indirect (cerebello-ponto-hypothalamic) pathways (Onat and Çavdar 2003; Çavdar et al. 2018; Kamali et al. 2018a, b, Bohne et al. 2019). The hypothalamic nuclei are also connected to the prefrontal cortex and other limbic nuclei, such as the septal nuclei and BNST via the PFCT (Figs. 5–8). The PFCT and MFB both contribute to the hypothalamo-prefrontal connectivity (Fig. 6). The hypothalamic nuclei are also tightly integrated with the amygdala via the amygdalofugal tract (AFT) (Figs. 4, 8) (Reppucci and Petrovich 2016). Unlike the amygdala, which is in contact with the prefrontal cortex via the uncinate fasciculus (UF) and cingulum bundle (CB), the hypothalamic nuclei communicate with the prefrontal cortex via the MFB and PFCT.

**Fig. 10:**
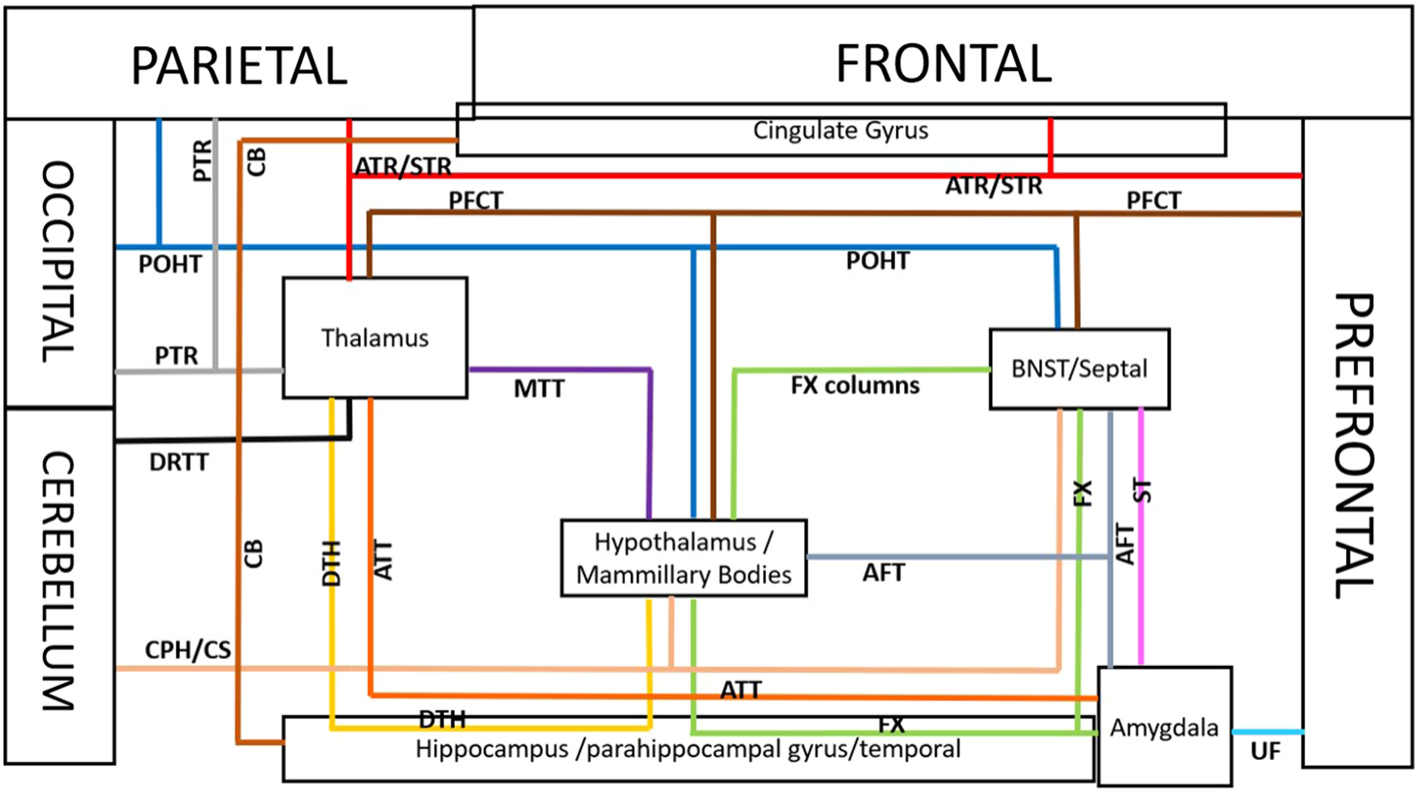
Schematic view of the major anterior (fronto-temporal connectivity) and posterior (parieto-occipito-cerebellar connectivity) neuronal circuits of the limbic system connected to the frontal, parietal, occipital and temporal cortices as well as cerebellar hemispheres. By introducing posterior limbic pathways such as POHT, cerebello-hypothalamic and cerebello-septal/BNST, DRTT and PTR, several additional neuronal circuits are added to the limbic network. This schematic view shows the tight connectivity of the limbic gray matter nuclei to the posterior cerebral and cerebellar cortices including the parietal, occipital lobes and cerebellar hemispheres. The circuits are detailed in the manuscript

The hypothalamus is at the center of multiple loops and circuits due to its central location and crucial role in many limbic functions. The hypothalamic nuclei are in connectivity with many limbic and nonlimbic structures and are a part of many loops and circuits, some of which are detailed below (Figs. 9 and 10).

$${\text{hypothalamus}}\, \Leftarrow \,\left( {{\text{MTT}}} \right)\, \Rightarrow \,{\text{thalamus}}\, \Leftarrow \,\left( {{\text{DTH}}} \right)\, \Rightarrow \,{\text{hippocampus}}\, \Leftarrow \,\left( {{\text{DTH}}} \right)\, \Rightarrow \,{\text{hypothalamus}}$$

$${\text{amygdala}}\, \Rightarrow \,\left( {{\text{AFT}}} \right)\, \Rightarrow \,{\text{hypotalamus/septal}}\,{\text{nuc}}\, \Leftarrow \,\left( {\text{PFCT/MFB}} \right)\, \Rightarrow \,{\text{prefrontal}}\,{\text{cortex}}\, \Leftarrow \,\left( {{\text{UF,}}\,{\text{CB}}} \right)\, \Rightarrow \,{\text{amygdala}}$$

$${\text{orbitofrontal/prefrontal/temporal}}\,{\text{cortex}}\, \Leftarrow \,\left( {{\text{UF,}}\,{\text{CB}}} \right)\, \Rightarrow \,{\text{amygdala}}\, \Rightarrow \,\left( {{\text{AFT}}} \right)\, \Rightarrow \,{\text{hypothalamus/septal}}\,{\text{nuc}}\, \Leftarrow \,\left( {{\text{PFCT}}} \right)\, \Rightarrow \,{\text{caudate/nuc}}\,{\text{accubmbens}}\, \Leftarrow \,\left( {{\text{PFCT}}} \right)\, \Rightarrow \,{\text{orbitofrontal/prefrontal}}\,{\text{cortex}}$$

$${\text{orbitofrontal/prefrontal/temporal}}\,{\text{cortex}}\, \Leftarrow \,\left( {{\text{UF,}}\,{\text{CB}}} \right)\, \Rightarrow \,{\text{amygdala}}\, \Rightarrow \,\left( {{\text{ATT}}} \right)\, \Rightarrow \,{\text{mediodorsal}}\,{\text{thalamus}}\, \Leftarrow \,{\text{(DTH)}}\, \Rightarrow \,{\text{hypotahalamus/septal}}\,{\text{nuc}}\, \Leftarrow \,\left( {{\text{PFCT}}} \right)\, \Rightarrow \,{\text{orbitofrontal/prefrontal}}\,{\text{cortex}}$$

$${\text{parieto - occipital}}\,{\text{cortex}}\, \Leftarrow \,\left( {{\text{POHT}}} \right)\, \Rightarrow \,{\text{hypothalamus/septal}}\,{\text{nuc}}\, \Leftarrow \,\left( {{\text{DTH}}} \right)\, \Rightarrow \,{\text{mediodorsal}}\,{\text{thalamus}}\, \Leftarrow \,\left( {{\text{PTR}}} \right)\, \Rightarrow \,{\text{parieto - occipital}}\,{\text{cortex}}$$

$${\text{parieto - occipital}}\,{\text{cortex}}\, \Leftarrow \,\left( {{\text{POHT}}} \right)\, \Rightarrow \,{\text{hypothamus/septal}}\,{\text{nuc}}\, \Leftarrow \,\left( {{\text{PFCT}}} \right)\, \Rightarrow \,{\text{orbitofrontal/prefrontal}}\, \Leftarrow \,\left( {{\text{CB}}} \right)\, \Rightarrow \,{\text{parieto - occipital}}\,{\text{cortex}}$$

### The Septal Nuclei

The septal nuclei are a band of gray matter nuclei located rostral to the anterior commissure, anterior to the lamina terminalis, and inferior to the rostrum of the corpus callosum extending alongside the lamina terminalis to the optic chiasm (Fig. 8C-D). Due to the close proximity of the hypothalamic and septal nuclei, almost all of the pathways connecting to the hypothalamic nuclei have some connectivity with septal nuclei as well (Haines 2007; Nolte 2009). The septal nuclei are also connected to the hypothalamic nuclei directly via the forniceal columns and stria terminalis (Fig. 3) (Rajmohan and Mohandas 2007, Catani et al. 2013, Kamali et al. 2015). Direct connectivity of the septal nuclei has also been shown with the amygdala, bed nucleus of stria terminalis (BNST), hypothalamus, and hippocampal formations (Fig. 7) (Haines 2007, Deng et al. 2019). Similar to the hypothalamus, the septal nuclei receive reciprocal connections from many other limbic and non-limbic structures such as the amygdala, cingulate gyrus, ventral tegmental area, hippocampus, thalamus and hypothalamus (Haines 2007; Nolte 2009). The septal nuclei send projections mainly to the habenular nuclei and the medial dorsal nucleus of the thalamus via the stria medullaris (Haines 2007; Nolte 2009). The septal nuclei are connected to the hippocampus and amygdala via the fornix and stria terminalis, and to the ventral tegmental area via the median forebrain bundle (Fig. 4) (Felten et al. 2015, Kamali et al. 2015). Direct connectivity of the septal nuclei to the amygdala was also described via the amygdalofugal tract (AFT) (Fig. 8). The AFT connects the amygdala to the septal nuclei, hypothalamic nuclei and BNST (Nolte 2009; Kamali et al. 2016). Moreover, the fornix connects the septal nuclei with the hippocampus, amygdala, and hypothalamic nuclei (Figs. 1, 3–4).

Direct connectivity of the septal nuclei with the cerebral cortex has been described, including the frontal, parietal, occipital, and temporal cortices (Haines 2007; Nolte 2009). The septal nuclei are connected to the prefrontal cortex via the prefronto-caudo-thalamic tract (PFCT) (Figs. 5–6) (Kamali et al. 2010). The thalamus is connected to the septal nuclei via the stria medullaris and PFCT (Fig. 5A, D) (Roman et al. 2020). A direct thalamo-septal connection was also reported via the fibers running side by side with the DTH (Kamali et al. 2020a, b). Direct connectivity of the septal nuclei with the parietal and occipital cortices was also shown along the course of the parieto-occipito-hypothalamic tract (POHT) (Swanson and Cowan 1979, Kamali et al. 2020b).

Connectivity of the septal nuclei with the cerebellar hemispheres was also described through the pons and middle cerebellar peduncles as part of the cerebello-ponto-hypothalamic tract (Stoodley and Schmahmann 2010; Kamali et al. 2020a, b).

Some of the circuits involving the hypothalamic and septal nuclei are detailed in the hypothalamus section and illustrated in Figs. 9–10. (Please attention that the anterior limbic circuits (fronto-temporal connectivity) are shown in Figs. 9 and the combination of the anterior and posterior limbic circuits (including the parieto-occipito-cerebellar connectivity) are shown in Figs. 10 for better clarification for readers).

### The Bed Nucleus of the Stria Terminalis (BNST)

The bed nucleus of stria terminalis is a collection of nuclei located at the base of the lateral ventricles (Fig. 6B). The BNST is located ventral to the septum, just above and posterior to the anterior commissure, and antero-superior to the hypothalamic nuclei (Fig. 8C) (Haines 2007).

Given the proximity of the BNST, septal and hypothalamic nuclei, they share many projections between one another (Nolte 2009). The study by Lebow showed that most of the projections reaching the hypothalamus were from the BNST (Lebow and Chen 2016). Similar to the hypothalamus and septal nuclei, the BNST has tight connectivity with the prefrontal cortex, amygdala, hippocampus, thalamus, basal ganglia, and brainstem nuclei/ventral tegmental area (Haines 2007).

The stria terminalis connects the BNST to the amygdala by a C-shaped course (Kamali et al. 2015; Dzafic et al. 2019; Hofmann and Straube 2021). Direct projections were also detected from the occipital cortex to the BNST in the human brain (Catani et al. 2003; Avery et al. 2014, Kamali et al.2020b). The large projection bundle of the prefronto-caudo-thalamic tract (PFCT) also has connectivity with the BNST (described later). The PFCT directly connects the thalamus to the BNST, hypothalamic and septal nuclei, then into the caudate nucleus and nucleus accumbens on its way to project into the prefrontal cortex. The BNST is also connected to the septal and hypothalamic nuclei via the forniceal columns and stria terminalis (Kamali et al. 2015). In the midline, some of the superior projections of the amygdalofugal tract project toward and terminate at the BNST, which provide a direct amygdalo-BNST connectivity (Fig. 8) (Weller and Smith 1982). By means of connectivity, the BNST may participate in many limbic circuits which involve both the hypothalamic and septal nuclei described earlier (Fig. 10).

$${\text{amygdala/hippocampus}}\, \Rightarrow \,\left( {{\text{AFT}}} \right)\, \Rightarrow \,{\text{BNST}}\, \Leftarrow \,\left( {{\text{PFCT}}} \right)\, \Rightarrow\,{\text{prefrontal}}\,{\text{cortex}}\, \Leftarrow \,\left( {{\text{UF,}}\,{\text{CB}}} \right)\, \Rightarrow \,{\text{amygdala/hippocampus}}$$

### The Parieto-Occipito-Hypothalamic Tract (POHT)

The POHT is a direct corticolimbic connectivity of the human hypothalamus connecting the parieto-occipital cornices directly to the hypothalamic nuclei (Kamali et al. 2020b). This pathway is one of the somatosensory and visual cortex-limbic system connectivity, and it may contribute to the cortico-limbo-cortical loop. Along its course toward the anterior hypothalamic nuclei, the POHT traverses through the nucleus basalis of Meynert. The POHT courses through the nucleus basalis alongside the ventral amygdalofugal tract. They both continue medially and project into the anterior hypothalamic nuclei. The POHT may provide a conduit for the visual and sensory information to both the nucleus basalis and anterior hypothalamic nuclei.

The POHT may contribute to multiple cortico-limbo-cortical circuits detailed in the hypothalamus section (Fig. 10).

### The Medial Forebrain Bundle (MFB)

The MFB is the most prominent mesolimbic reward pathway connecting the ventral tegmental areas with the prefrontal cortex (Nolte 2009). Two subsegments of the MFB have been described by Coenen et al. known as the inferomedial and superolateral subsegments (Coenen et al. 2012, Cho et al. 2015). The MFB connects several brain regions both rostrally and caudally, including the periaqueductal gray matter in the mesencephalon to the lateral hypothalamic nuclei and prefrontal cortex (Fig. 7). The MFB is a long projection bundle arising from the posterior aspect of the cervico-medullary junction and ascending through the posterior part of the brainstem to the ventral tegmental area in the midbrain (Nolte 2009). The MFB then projects from the ventral tegmental areas of the midbrain into the prefrontal cortex via the anterior limbs of the internal capsules (Fig. 7). The MFB courses near the midline in the midbrain from the periaqueductal gray matter toward the ventral tegmental areas and then projects laterally along the walls of the interpeduncular cistern away from the third ventricle, toward the lateral hypothalamic nuclei (Fig. 7A). The MFB then projects superiorly into the internal capsule at the genu and courses anteriorly toward the prefrontal cortex within the anterior limb of the internal capsule (Fig. 7C-D). More distally within the anterior limb of the internal capsule, the MFB splits into the medial and lateral projections, which project into the medial and lateral prefrontal cortices (Fig. 7).

The PFCT courses outside and medially in respect to the anterior limb of the internal capsule through the caudate head. The MFB plays a part in multiple limbic circuits as the prefronto-hypothalamic/septal connecting arm (Fig. 9).

$${\text{prefrontal}}\,{\text{cortex}}\, \Leftarrow \,\left( {{\text{UF,}}\,{\text{CB}}} \right)\, \Rightarrow \,{\text{amygdala}}\, \Rightarrow \,\left( {{\text{AFT}}} \right)\, \Rightarrow \,{\text{hypothalamus/septal}}\,{\text{nuc}}\, \Leftarrow \,\left( {{\text{MFB}}} \right)\, \Rightarrow \,{\text{prefrontal}}\,{\text{cortex}}$$

$${\text{prefrontal}}\,{\text{cortex}}\, \Leftarrow \,{\text{(PFCT)}}\, \Rightarrow \,{\text{ventral}}\,{\text{striatum}}\, \Leftarrow \,\left( {{\text{mesolimbic}}\,{\text{pathway}}} \right)\, \Rightarrow \,{\text{ventral}}\,{\text{tegmental}}\,{\text{area}}\, \Leftarrow \left( {{\text{MFB}}} \right)\, \Rightarrow \,{\text{prefrontal}}\,{\text{cortex}}$$

### The Basal Ganglia (BG)

The basal ganglia nuclei (BG) consist of multiple gray matter nuclei of the diencephalon in the basal forebrain, tightly connected to the cerebral cortex, thalamus, and brainstem nuclei (Haines 2007; Nolte 2009; Stephenson-Jones et al. 2016). The basal ganglia include the ventral striatum (e.g., olfactory tubercle, anterior perforated substance, nucleus accumbens), globus pallidus, ventral pallidum, and substantia nigra (Nolte 2009). The combination of the putamen and globus pallidus is called the lentiform nuclei (Nolte 2009). The combination of the caudate and putamina is called striatum (Nolte 2009). The ventral striatum and dorsal striatum may serve as the input nuclei in the circuits involving the basal ganglia (Haber 2016). They accept signals from the cerebral cortex, mesencephalic/nigral nuclei, and thalamus. The BG's output nuclei are the globus pallidus interna and the substantia nigra pars reticulata, which send impulses to the thalamus and cortex (Haber 2016). The frontostriatal fibers are part of the PFCT as described before.

The cortico-basal ganglia-thalamo-cortical pathway is another loop including the thalamus, limbic and basal ganglia. The cortico-basal ganglia-thalamo-cortical loops are thought to have consisted of parallel circuits that individually process limbic, associative, and sensorimotor information (Haber 2016; Fazl and Fleisher 2018; Aoki et al. 2019). Other BG nuclei include the subthalamic nucleus, the globus pallidus externa, and the substantia nigra pars compacta (Haber 2016; Fazl and Fleisher 2018; Aoki et al. 2019). Multiple small fascicles connecting the basal ganglia with the brainstem nuclei are recently revealed by Oishi et al. 2020 (Oishi et al. 2020). The anatomy of the Claustrum-Cortex Interactions was also demonstrated by Jackson et al. 2020 (Jackson et al. 2020).

### The Nucleus Accumbens (NAC)

The NAC is a gray matter structure of the ventral striatum located at the junction of the anterior caudate and putamina in the anterior-most part of the internal capsule, just slightly posterior and inferior to the septal nuclei (Fig. 6C) (Salgado and Kaplitt 2015). A large projection bundle was described as the prefronto-caudo-thalamic tract (PFCT), coursing from the thalamus to the caudate and NAC and then into the medial aspect of the prefrontal cortex (Fig. 6) (Robinson and Kolb 1997; Kamali et al. 2010). The nucleus accumbens also receives projections from the ventral tegmental area (Ikemoto 2007).

Efferent pathways have also been described projecting from the NAC into several limbic structures, including the stria terminalis, preoptic and lateral hypothalamic nuclei, amygdala, thalamus, and cingulum (Salgado and Kaplitt 2015). Given the connections between the NAC with both the limbic system and motor nuclei of the basal ganglia, the NAC may play a role in communication between limbic and motor systems (Robinson and Kolb 1997).

### The Nucleus Basalis of Meynert (NBM)

The nucleus basalis is the largest collection of cholinergic neurons in the brain, located in the substantia innominate of the basal forebrain, inferior to the anterior commissure and the globus pallidus, and lateral to the anterior hypothalamus (Fig. 8A-B) (Haines 2007). Cholinergic neurons of the basal forebrain, including the basal nucleus of Meynert, are known to be involved in memory function (Nolte 2009; Liu et al. 2015; Li et al. 2017). The NBM may modulate the activity of the neocortex, including the limbic areas, with widespread cholinergic cortical projections. The NBM has connections with other brain structures such as the lateral hypothalamus, amygdala, and midbrain. The degenerative process in the basal nucleus of Meynert may play a central role in memory impairments in degenerative disorders such as Alzheimer’s and Parkinson’s diseases (Liu et al. 2015). Disproportionate atrophy of the parietal and temporal lobes is described in Alzheimer’s disease. The POHT and amygdalofugal tracts run in the parietal and temporal lobes, and both course through the NBM before projecting into the hypothalamic nuclei. Therefore, these two pathways may be affected in cognitive and memory dysfunction in Alzheimer’s disease.

## Conclusions and Future Directions

In this review, we included the most recent findings of diffusion weighted tractography studies. Our novel contribution to the understanding of the circuits as presented in this manuscript is by stepwise introduction of the newly traced limbic fiber tracts and inclusion of the possible circuits they may generate or be a part of in the limbic system. We elaborated on the role of the new fibers in adding multiple new neuronal loops and circuits to the previously known limbic models. The proposed model could potentially help clinicians and neuroscientists to detect possible correlations between symptoms and impaired regions of the limbic network. Future studies are required to confirm and expand the anatomy of these tracts and their involvement in neuropsychiatric disorders.

## Supplementary Information

Below is the link to the electronic supplementary material.

Supplementary file1 (PPTX 203 KB)

## Abbreviations

AC: Anterior commissure
AD: Alzheimer’s disease
AFT: Amygdalofugal tract
ALIC: Anterior limb of the internal capsule
ATR: Anterior thalamic radiation
ATT: Amygdalothalamic tract
BG: Basal ganglia
BNST: Bed nucleus of the stria terminalis
CB: Cingulum bundle
CPH: Cerebello-ponto-hypothalamic tract
DBB: Diagonal Band of Broca
DM: Dorsomedial nucleus of the thalamus
DRTT: Dentato-rubro-thalamic tract
DTH: Dorsal thalamo-hypothalamic tract
DTI: Diffusion tensor imaging
DWI: Diffusion-weighted imaging
FX: Fornix
MFB: Medial forebrain bundle
MTT: Mammillothalamic tract
NAC: Nucleus accumbens
MB: Mammillary body
NBM: Nucleus basalis of Meynert
PFC: Prefrontal cortex
PFCT: Prefronto-caudo-thalamic tract
POHT: Parieto-occipito-hypothalamic tract
PTR: Posterior thalamic radiation
ST: Stria terminalis
STR: Superior thalamic radiation
TC: Thalamo-cingulate tract
UF: Uncinate fasciculus

## References

[CR1] Aggleton, J. and M. Mishkin (1982). A comparison of amygdaloid and hippocampal projections to the thalamus in monkeys. SOC. Neurosci. Abst 8(240.6).

[CR2] Aggleton JP, O’Mara SM, Vann SD, Wright NF, Tsanov M, Erichsen JT (2010). Hippocampal–anterior thalamic pathways for memory: uncovering a network of direct and indirect actions. Eur J Neurosci.

[CR3] Al-Chalabi, M. and S. Gupta (2018). Neuroanatomy, spinothalamic tract. 29939601

[CR4] Aoki S, Smith JB, Li H, Yan X, Igarashi M, Coulon P, Wickens JR, Ruigrok TJ, Jin X (2019). An open cortico-basal ganglia loop allows limbic control over motor output via the nigrothalamic pathway. Elife.

[CR5] Avery SN, Clauss JA, Winder DG, Woodward N, Heckers S, Blackford JU (2014). BNST neurocircuitry in humans. Neuroimage.

[CR6] Barbas H, Saha S, Rempel-Clower N, Ghashghaei T (2003). Serial pathways from primate prefrontal cortex to autonomic areas may influence emotional expression. BMC Neurosci.

[CR7] Benagiano V, Rizzi A, Lorusso L, Flace P, Saccia M, Cagiano R, Ribatti D, Roncali L, Ambrosi G (2018). The functional anatomy of the cerebrocerebellar circuit: a review and new concepts. J Comp Neurol.

[CR8] Bocchio M, McHugh SB, Bannerman DM, Sharp T, Capogna M (2016). Serotonin, amygdala and fear: assembling the puzzle. Front Neural Circuits.

[CR9] Bohne P, Schwarz MK, Herlitze S, Mark MD (2019). A new projection from the deep cerebellar nuclei to the hippocampus via the ventrolateral and laterodorsal thalamus in mice. Front Neural Circuits.

[CR10] Broca P (1878). Anatomie comparée des circonvolutions cérébrales. Le grand lobe limbique et la scissure limbique dans la série des mammifères. Rev Anthrop.

[CR11] Bryant KL, Li L, Eichert N, Mars RB (2020). A comprehensive atlas of white matter tracts in the chimpanzee. PLoS Biol.

[CR12] Catani M, Jones DK, Donato R, Ffytche DH (2003). Occipito-temporal connections in the human brain. Brain.

[CR13] Catani M, Dell’Acqua F, De Schotten MT (2013). A revised limbic system model for memory, emotion and behaviour. Neurosci Biobehav Rev.

[CR14] Çavdar S, Özgur M, Kuvvet Y, Bay HH (2018). The cerebello-hypothalamic and hypothalamo-cerebellar pathways via superior and middle cerebellar peduncle in the rat. Cerebellum.

[CR15] Chen S, Su H-S (1990). Afferent connections of the thalamic paraventricular and parataenial nuclei in the rat—a retrograde tracing study with iontophoretic application of Fluoro-Gold. Brain Res.

[CR16] Cho Z-H, Law M, Chi J-G, Choi S-H, Park S-Y, Kammen A, Park C-W, Oh S-H, Kim Y-B (2015). An anatomic review of thalamolimbic fiber tractography ultra-high resolution direct visualization of thalamolimbic fibers anterior thalamic radiation, superolateral and inferomedial medial forebrain bundles and newly identified septum pellucidum tract. World Neurosurg.

[CR17] Coenen VA, Panksepp J, Hurwitz TA, Urbach H, Mädler B (2012). Human medial forebrain bundle (MFB) and anterior thalamic radiation (ATR): imaging of two major subcortical pathways and the dynamic balance of opposite affects in understanding depression. J Neuropsychiatry Clin Neurosci.

[CR18] Conrad LC, Pfaff DW (1976). Efferents from medial basal forebrain and hypothalamus in the rat. I. An autoradiographic study of the medial preoptic area. J Comp Neurol.

[CR19] Cornwall J, Phillipson O (1988). Afferent projections to the dorsal thalamus of the rat as shown by retrograde lectin transport. II. The midline nuclei. Brain Res Bull.

[CR20] Crosby EC (1963). Correlative anatomy of the nervous system. Acad Med.

[CR21] Davidson S, Zhang X, Khasabov SG, Simone DA, Giesler GJ (2008). Termination zones of functionally characterized spinothalamic tract neurons within the primate posterior thalamus. J Neurophysiol.

[CR22] Davis M, Whalen PJ (2001). The amygdala: vigilance and emotion. Mol Psychiatry.

[CR23] Deng K, Yang L, Xie J, Tang H, Wu G-S, Luo H-R (2019). Whole-brain mapping of projection from mouse lateral septal nucleus. Biol Open..

[CR24] Di X, Huang J, Biswal BB (2017). Task modulated brain connectivity of the amygdala: a meta-analysis of psychophysiological interactions. Brain Struct Funct.

[CR25] Dillingham CM, Frizzati A, Nelson AJ, Vann SD (2015). How do mammillary body inputs contribute to anterior thalamic function?. Neurosci Biobehav Rev.

[CR26] Dillingham CM, Milczarek MM, Perry JC, Vann SD (2020). Time to put the mammillothalamic pathway into context. Neurosci Biobehavioral Rev.

[CR27] Dzafic I, Oestreich L, Martin AK, Mowry B, Burianová H (2019). Stria terminalis, amygdala, and temporoparietal junction networks facilitate efficient emotion processing under expectations. Hum Brain Mapp.

[CR28] Ebeling U, Cramon DV (1992). Topography of the uncinate fascicle and adjacent temporal fiber tracts. Acta Neurochir.

[CR29] Fama R, Sullivan EV (2015). Thalamic structures and associated cognitive functions: relations with age and aging. Neurosci Biobehav Rev.

[CR30] Fazl A, Fleisher J (2018). Anatomy, physiology, and clinical syndromes of the basal ganglia: a brief review. Semin Pediatr Neurol.

[CR31] Felten DL, O'Banion MK, Maida ME (2015). Netter's atlas of neuroscience.

[CR32] Gallagher M, Holland PC (1994). The amygdala complex: multiple roles in associative learning and attention. Proc Natl Acad Sci.

[CR33] George K, Das JM (2020). Neuroanatomy, thalamocortical radiations.

[CR34] Ghashghaei H, Barbas H (2002). Pathways for emotion: interactions of prefrontal and anterior temporal pathways in the amygdala of the rhesus monkey. Neuroscience.

[CR35] Grodd W, Kumar VJ, Schüz A, Lindig T, Scheffler K (2020). The anterior and medial thalamic nuclei and the human limbic system: tracing the structural connectivity using diffusion-weighted imaging. Sci Rep.

[CR36] Groenewegen H (1988). Organization of the afferent connections of the mediodorsal thalamic nucleus in the rat, related to the mediodorsal-prefrontal topography. Neuroscience.

[CR37] Haber SN (2016). Corticostriatal circuitry. Dialogues Clin Neurosci.

[CR38] Haber SN, Kunishio K, Mizobuchi M, Lynd-Balta E (1995). The orbital and medial prefrontal circuit through the primate basal ganglia. J Neurosci.

[CR39] Haines DE (2007). Neuroanatomy: an atlas of structures, sections, and systems.

[CR40] Haller J (2018). The role of central and medial amygdala in normal and abnormal aggression: a review of classical approaches. Neurosci Biobehav Rev.

[CR41] Herkenham M (1978). The connections of the nucleus reuniens thalami: evidence for a direct thalamo-hippocampal pathway in the rat. J Comp Neurol.

[CR42] Hofmann D, Straube T (2021). Effective connectivity between bed nucleus of the stria terminalis and amygdala: reproducibility and relation to anxiety. Hum Brain Mapp.

[CR43] Ikemoto S (2007). Circuito de recompensa de dopamina: dos sistemas de proyección desde el cerebro medio ventral al núcleo accumbens-complejo tuberculoso olfativo. Brain Res Rdo.

[CR44] Jackson J, Smith JB, Lee AK (2020). The anatomy and physiology of claustrum-cortex interactions. Annu Rev Neurosci.

[CR45] Jakob C (1906). Nueva contribución á la fisio-patología de los lóbulos frontales. La Semana Médica.

[CR46] Jang SH, Hong JH (2012). The anatomical characteristics of superior longitudinal fasciculus I in human brain: diffusion tensor tractography study. Neurosci Lett.

[CR47] Jang SH, Yeo SS (2013). Thalamocortical tract between anterior thalamic nuclei and cingulate gyrus in the human brain: diffusion tensor tractography study. Brain Imaging Behav.

[CR48] Jang S, Yeo SS (2014). Thalamocortical connections between the mediodorsal nucleus of the thalamus and prefrontal cortex in the human brain: a diffusion tensor tractographic study. Yonsei Med J.

[CR49] Jellison BJ, Field AS, Medow J, Lazar M, Salamat MS, Alexander AL (2004). Diffusion tensor imaging of cerebral white matter: a pictorial review of physics, fiber tract anatomy, and tumor imaging patterns. Am J Neuroradiol.

[CR50] Jones EG (2012). The thalamus.

[CR51] Kahle W, Platzer W, Frotscher M, Leonhardt H (2002). Color Atlas and Textbook of Human Anatomy Thieme.

[CR52] Kaitz SS, Robertson RT (1981). Thalamic connections with limbic cortex. II. Corticothalamic projections. J Comp Neurol.

[CR53] Kamali A, Kramer LA, Butler IJ, Hasan KM (2009). Diffusion tensor tractography of the somatosensory system in the human brainstem: initial findings using high isotropic spatial resolution at 3.0 T. Eur Radiol.

[CR54] Kamali A, Kramer LA, Hasan KM (2010). Feasibility of prefronto-caudate pathway tractography using high resolution diffusion tensor tractography data at 3 T. J Neurosci Methods.

[CR55] Kamali A, Hasan KM, Adapa P, Razmandi A, Keser Z, Lincoln J, Kramer LA (2014). Distinguishing and quantification of the human visual pathways using high-spatial-resolution diffusion tensor tractography. Magn Reson Imaging.

[CR56] Kamali A, Yousem DM, Lin DD, Sair HI, Jasti SP, Keser Z, Riascos RF, Hasan KM (2015). Mapping the trajectory of the stria terminalis of the human limbic system using high spatial resolution diffusion tensor tractography. Neurosci Lett.

[CR57] Kamali A, Sair HI, Blitz AM, Riascos RF, Mirbagheri S, Keser Z, Hasan KM (2016). Revealing the ventral amygdalofugal pathway of the human limbic system using high spatial resolution diffusion tensor tractography. Brain Struct Funct.

[CR58] Kamali A, Riascos RF, Pillai JJ, Sair HI, Patel R, Nelson FM, Lincoln JA, Tandon N, Mirbagheri S, Rabiei P (2018). Mapping the trajectory of the amygdalothalamic tract in the human brain. J Neurosci Res.

[CR59] Kamali A, Zhang CC, Riascos RF, Tandon N, Bonafante-Mejia EE, Patel R, Lincoln JA, Rabiei P, Ocasio L, Younes K (2018). Diffusion tensor tractography of the mammillothalamic tract in the human brain using a high spatial resolution DTI technique. Sci Rep.

[CR60] Kamali A, Karbasian N, Sherbaf FG, Wilken LA, Aein A, Sair HI, Espejo OA, Rabiei P, Choi SJ, Mirbagheri S (2020). Uncovering the dorsal thalamo-hypothalamic tract of the human limbic system. Neuroscience.

[CR62] Kamali A, Sherbaf FG, Rahmani F, Khayat-Khoei M, Aein A, Gandhi A, Shah EG, Sair HI, Riascos RF, Esquenazi Y (2020). A direct visuosensory cortical connectivity of the human limbic system. Dissecting the trajectory of the parieto-occipito-hypothalamic tract in the human brain using diffusion weighted tractography. Neurosci Lett.

[CR63] Keser Z, Meier EL, Stockbridge MD, Breining BL, Sebastian R, Hillis AE (2021). Thalamic nuclei and thalamocortical pathways after left hemispheric stroke and their association with picture naming. Brain Connect.

[CR64] Kier EL, Staib LH, Davis LM, Bronen RA (2004). MR imaging of the temporal stem: anatomic dissection tractography of the uncinate fasciculus, inferior occipitofrontal fasciculus, and Meyer’s loop of the optic radiation. Am J Neuroradiol.

[CR65] Kiernan J (2012). Anatomy of the temporal lobe. Epilepsy Res Treat.

[CR66] Klingler J, Gloor P (1960). The connections of the amygdala and of the anterior temporal cortex in the human brain. J Comp Neurol.

[CR67] Krout KE, Loewy AD (2000). Parabrachial nucleus projections to midline and intralaminar thalamic nuclei of the rat. J Comp Neurol.

[CR68] Lambert C, Simon H, Colman J, Barrick TR (2017). Defining thalamic nuclei and topographic connectivity gradients in vivo. Neuroimage.

[CR69] Lawes INC, Barrick TR, Murugam V, Spierings N, Evans DR, Song M, Clark CA (2008). Atlas-based segmentation of white matter tracts of the human brain using diffusion tensor tractography and comparison with classical dissection. Neuroimage.

[CR70] Lebow MA, Chen A (2016). Overshadowed by the amygdala: the bed nucleus of the stria terminalis emerges as key to psychiatric disorders. Mol Psychiatry.

[CR71] Lemaire J-J, Nezzar H, Sakka L, Boirie Y, Fontaine D, Coste A, Coll G, Sontheimer A, Sarret C, Gabrillargues J (2013). Maps of the adult human hypothalamus. Surg Neurol Int.

[CR72] Li H, Jia X, Qi Z, Fan X, Ma T, Ni H, Li C-SR, Li K (2017). Altered functional connectivity of the basal nucleus of Meynert in mild cognitive impairment: a resting-state fMRI study. Front Aging Neurosci.

[CR73] Liu AKL, Chang RC-C, Pearce RK, Gentleman SM (2015). Nucleus basalis of Meynert revisited: anatomy, history and differential involvement in Alzheimer’s and Parkinson’s disease. Acta Neuropathol.

[CR74] Lövblad K-O, Schaller K, Vargas MI (2014). The fornix and limbic system Seminars in Ultrasound CT and MRI.

[CR75] MacLean PD (1949). Psychosomatic disease and the" visceral brain"; recent developments bearing on the Papez theory of emotion. Psychosom Med.

[CR76] MacLean PD (1952). Some psychiatric implications of physiological studies on frontotemporal portion of limbic system (visceral brain). Electroencephalogr. Clin. Neurophysiol..

[CR77] Mamah D, Conturo TE, Harms MP, Akbudak E, Wang L, McMichael AR, Gado MH, Barch DM, Csernansky JG (2010). Anterior thalamic radiation integrity in schizophrenia: a diffusion-tensor imaging study. Psychiatr Res: Neuroimaging.

[CR78] Mandelbaum G, Taranda J, Haynes TM, Hochbaum DR, Huang KW, Hyun M, Venkataraju KU, Straub C, Wang W, Robertson K (2019). Distinct cortical-thalamic-striatal circuits through the parafascicular nucleus. Neuron.

[CR79] Mori S, Kageyama Y, Hou Z, Aggarwal M, Patel J, Brown T, Miller MI, Wu D, Troncoso JC (2017). Elucidation of white matter tracts of the human amygdala by detailed comparison between high-resolution postmortem magnetic resonance imaging and histology. Front Neuroanat.

[CR80] Nakano I (1998). The limbic system: an outline and brief history of its concept. Neuropathology.

[CR81] Nauta WJ, Domesick VB (1978). Crossroads of limbic and striatal circuitry: hypothalamo-nigral connections.

[CR82] Nguyen T-V, Gower P, Albaugh MD, Botteron KN, Hudziak JJ, Fonov VS, Collins L, Ducharme S, McCracken JT (2016). The developmental relationship between DHEA and visual attention is mediated by structural plasticity of cortico-amygdalar networks. Psychoneuroendocrinology.

[CR83] Nieuwenhuys R, Voogd J, van Huijzen C (1988). Vessels and Meninges.

[CR84] Noback CR, Ruggiero DA, Demarest RJ, Strominger NL (2005). The human nervous system: structure and function.

[CR85] Nolte J (2009). The Human Brain: An Introduction to It's Functional Anatomy.

[CR86] Nooij R, Hoving E, van Hulzen A, Cornelissen FW, Renken R (2015). Preservation of the optic radiations based on comparative analysis of diffusion tensor imaging tractography and anatomical dissection. Front Neuroanat.

[CR87] Oishi K, Mori S, Troncoso JC, Lenz FA (2020). Mapping tracts in the human subthalamic area by 11.7 T ex vivo diffusion tensor imaging. Brain Struct Funct.

[CR88] Onat F, Çavdar S (2003). Cerebellar connections: hypothalamus. Cerebellum.

[CR89] Otake K, Reis DJ, Ruggiero DA (1994). Afferents to the midline thalamus issue collaterals to the nucleus tractus solitarii: an anatomical basis for thalamic and visceral reflex integration. J Neurosci.

[CR90] Papez JW (1937). A proposed mechanism of emotion. Arch Neurol Psychiatry.

[CR91] Pascalau R, Stănilă RP, Sfrângeu S, Szabo B (2018). Anatomy of the limbic white matter tracts as revealed by fiber dissection and tractography. World Neurosurgery.

[CR92] Pasquier DA, Reinoso-Suarez F (1976). Direct projections from hypothalamus to hippocampus in the rat demonstrated by retrograde transport of horseradish peroxidase. Brain Res.

[CR93] Peterson DC, Reddy V, Mayes DA (2019) Neuroanatomy, mammillary bodies 30725877

[CR94] Rajmohan V, Mohandas E (2007). The limbic system. Indian J Psychiatr.

[CR95] Rapan L, Froudist-Walsh S, Niu M, Xu T, Funck T, Zilles K, Palomero-Gallagher N (2021). Multimodal 3D atlas of the macaque monkey motor and premotor cortex. Neuroimage.

[CR96] Reppucci CJ, Petrovich GD (2016). Organization of connections between the amygdala, medial prefrontal cortex, and lateral hypothalamus: a single and double retrograde tracing study in rats. Brain Struct Funct.

[CR97] Ressler RL, Maren S (2019). Synaptic encoding of fear memories in the amygdala. Curr Opin Neurobiol.

[CR98] Robertson RT, Kaitz SS (1981). Thalamic connections with limbic cortex. I. Thalamocortical projections. J Comp Neurol.

[CR99] Robinson TE, Kolb B (1997). Persistent structural modifications in nucleus accumbens and prefrontal cortex neurons produced by previous experience with amphetamine. J Neurosci.

[CR100] Roman E, Weininger J, Lim B, Roman M, Barry D, Tierney P, O’Hanlon E, Levins K, O’Keane V, Roddy D (2020). Untangling the dorsal diencephalic conduction system: a review of structure and function of the stria medullaris, habenula and fasciculus retroflexus. Brain Struct Funct.

[CR101] Roxo MR, Franceschini PR, Zubaran C, Kleber FD, Sander JW (2011). The limbic system conception and its historical evolution. ScientificWorldJournal.

[CR102] Rudzinskas SA, Williams KM, Mong JA, Holder MK (2019). Sex, drugs, and the medial amygdala: a model of enhanced sexual motivation in the female rat. Front Behav Neurosci.

[CR103] Salgado S, Kaplitt MG (2015). The nucleus accumbens: a comprehensive review. Stereotact Funct Neurosurg.

[CR104] Schmahmann JD, Pandya DN, Wang R, Dai G, D'Arceuil HE, de Crespigny AJ, Wedeen VJ (2007). Association fibre pathways of the brain: parallel observations from diffusion spectrum imaging and autoradiography. Brain.

[CR105] Schmahmann JD, Smith EE, Eichler FS, Filley CM (2008). Cerebral white matter: neuroanatomy, clinical neurology, and neurobehavioral correlates. Ann N Y Acad Sci.

[CR106] Sherbondy AJ, Dougherty RF, Napel S, Wandell BA (2008). Identifying the human optic radiation using diffusion imaging and fiber tractography. J vis.

[CR107] Stephenson-Jones M, Yu K, Ahrens S, Tucciarone JM, van Huijstee AN, Mejia LA, Penzo MA, Tai L-H, Wilbrecht L, Li B (2016). A basal ganglia circuit for evaluating action outcomes. Nature.

[CR108] Stoodley CJ, Schmahmann JD (2010). Evidence for topographic organization in the cerebellum of motor control versus cognitive and affective processing. Cortex.

[CR109] Sun C, Wang Y, Cui R, Wu C, Li X, Bao Y, Wang Y (2018). Human thalamic-prefrontal peduncle connectivity revealed by diffusion spectrum imaging fiber tracking. Front Neuroanat.

[CR110] Sutherland RJ, Rodriguez A (1989). The role of the fornix/fimbria and some related subcortical structures in place learning and memory. Behav Brain Res.

[CR111] Swanson L (1976). An autoradiographic study of the efferent connections of the preoptic region in the rat. J Comp Neurol.

[CR112] Swanson LW, Cowan WM (1979). The connections of the septal region in the rat. J Comp Neurol.

[CR113] Ungerleider L, Mishkin M, Ingle D, Goodale M, Mansfield R (1982). Analysis of visual behavior.

[CR114] Usunoff KG, Schmitt O, Itzev DE, Haas SJ-P, Lazarov NE, Rolfs A, Wree A (2009). Efferent projections of the anterior and posterodorsal regions of the medial nucleus of the amygdala in the mouse. Cells Tissues Organs.

[CR115] Von Der Heide RJ, Skipper LM, Klobusicky E, Olson IR (2013). Dissecting the uncinate fasciculus: disorders, controversies and a hypothesis. Brain.

[CR116] Wakana S, Jiang H, Nagae-Poetscher LM, Van Zijl PC, Mori S (2004). Fiber tract–based atlas of human white matter anatomy. Radiology.

[CR117] Walf AA, Frye CA (2006). A review and update of mechanisms of estrogen in the hippocampus and amygdala for anxiety and depression behavior. Neuropsychopharmacology.

[CR118] Wang X, Pathak S, Stefaneanu L, Yeh F-C, Li S, Fernandez-Miranda JC (2016). Subcomponents and connectivity of the superior longitudinal fasciculus in the human brain. Brain Struct Funct.

[CR119] Weller K, Smith D (1982). Afferent connections to the bed nucleus of the stria terminalis. Brain Res.

[CR120] Whalen PJ, Rauch SL, Etcoff NL, McInerney SC, Lee MB, Jenike MA (1998). Masked presentations of emotional facial expressions modulate amygdala activity without explicit knowledge. J Neurosci.

[CR121] Wilkinson M, Kane T, Wang R, Takahashi E (2017). Migration pathways of thalamic neurons and development of thalamocortical connections in humans revealed by diffusion MR tractography. Cereb Cortex.

[CR122] Willis T., (1664) Cerebri Anatome. London: Martyn & Allestry

[CR123] Yakovlev PI (1948). Motility, behavior and the brain; stereodynamic organization and neural co-ordinates of behavior. J Nerv Mental Dis.

[CR124] Yamada K, Shrier DA, Rubio A, Yoshiura T, Iwanaga S, Shibata DK, Patel U, Numaguchi Y (1998). MR imaging of the mamillothalamic tract. Radiology.

[CR135] Zhou S-Y, Suzuki M, Hagino H, Takahashi T, Kawasaki Y, Nohara S, Yamashita I, Seto H, Kurachi M (2003). Decreased volume and increased asymmetry of the anterior limb of the internal capsule in patients with schizophrenia. Biol Psychiat.

[CR125] Zinn CG, Clairis N, Cavalcante LES, Furini CRG, de Carvalho Myskiw J, Izquierdo I (2016). Major neurotransmitter systems in dorsal hippocampus and basolateral amygdala control social recognition memory. Proc Natl Acad Sci.

