# Supplementary figures and images for: The Cortico-Limbo-Thalamo-Cortical Circuits: An Update to the Original Papez Circuit of the Human Limbic System
Source: Brain Topogr. 2023 Apr 26;36(3):371–89. doi: 10.1007/s10548-023-00955-y (PMC10164017; doi:10.1007/s10548-023-00955-y)

## Slide 1
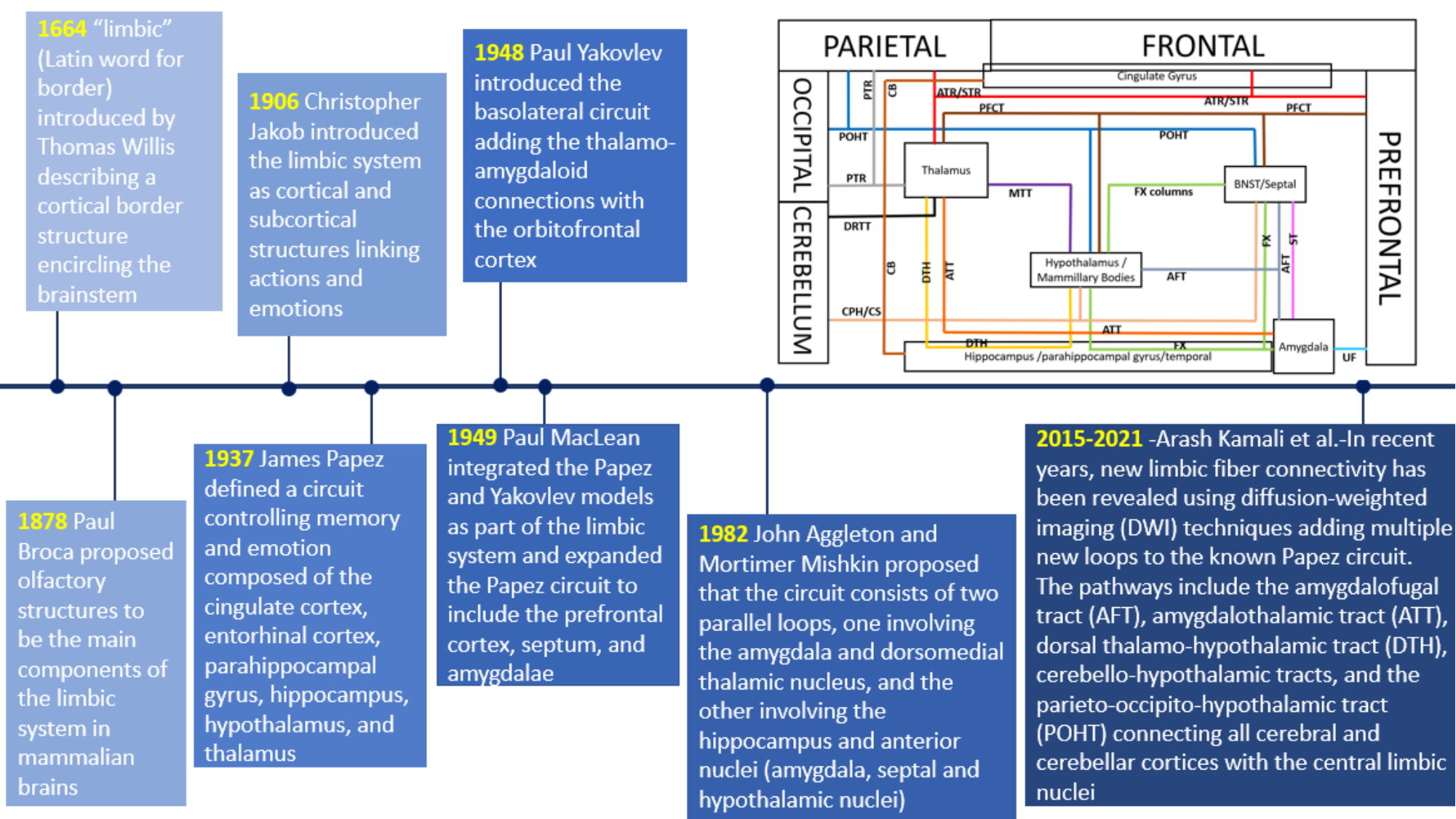

Supplement: Supplementary file 1 — Supplementary file1 (PPTX 203 KB) [file 10548_2023_955_MOESM1_ESM.pptx]
